# Sex- and age-dependent contribution of System x_*c*_^–^ to cognitive, sensory, and social behaviors revealed by comprehensive behavioral analyses of System x_*c*_^–^ null mice

**DOI:** 10.3389/fnbeh.2023.1238349

**Published:** 2023-08-15

**Authors:** Carla Frare, Shannon K. Pitt, Sandra J. Hewett

**Affiliations:** Department of Biology, Program in Neuroscience, Syracuse University, Syracuse, NY, United States

**Keywords:** spatial memory, recognition memory, anxiety, motor function, sensorimotor gating, C3H/HeSnJ, autism spectrum disorder, attention deficit hyperactivity disorder

## Abstract

**Background:**

System x_c_^–^ (Sx_c_^–^) is an important heteromeric amino acid cystine/glutamate exchanger that plays a pivotal role in the CNS by importing cystine into cells while exporting glutamate. Although certain behaviors have been identified as altered in Sx_c_^–^ null mutant mice, our understanding of the comprehensive impact of Sx_c_^–^ on behavior remains incomplete.

**Methods:**

To address this gap, we compared motor, sensory and social behaviors of male and female mice in mice null for Sx_c_^–^ (SLC7A11^sut/sut^) with wildtype littermates (SLC7A11^+/+^) in a comprehensive and systematic manner to determine effects of genotype, sex, age, and their potential interactions.

**Results:**

Motor performance was not affected by loss of Sx_c_^–^ in both males and females, although it was impacted negatively by age. Motor learning was specifically disrupted in female mice lacking Sx_c_^–^ at both 2 and 6 months of age. Further, female SLC7A11^sut/sut^ mice at both ages exhibited impaired sociability, but normal spatial and recognition memory, as well as sensorimotor gating. Finally, pronounced open-space anxiety was displayed by female SLC7A11^sut/sut^ when they were young. In contrast, young SLC7A11^sut/sut^ male mice demonstrated normal sociability, delayed spatial learning, increased open-space anxiety and heightened sensitivity to noise. As they aged, anxiety and noise sensitivity abated but hyperactivity emerged.

**Discussion:**

We find that the behavioral phenotypes of female SLC7A11^sut/sut^ are similar to those observed in mouse models of autism spectrum disorder, while behaviors of male SLC7A11^sut/sut^ resemble those seen in mouse models of attention deficit hyperactivity disorder. These results underscore the need for further investigation of *SLC7A11* in neurodevelopment. By expanding our understanding of the potential involvement of Sx_c_^–^, we may gain additional insights into the mechanisms underlying complex neurodevelopmental conditions.

## 1. Introduction

System x_c_^–^ (Sx_c_^–^) is a heteromeric amino acid cystine/glutamate exchanger that under physiological conditions imports L-cystine into cells while countertransporting L-glutamate with 1:1 stoichiometry (Bannai and Kitamura, 1980; Bannai, 1986). Tissue expression studies in rodents demonstrate high expression of *SLC7A11* — which encodes for substrate-specific light chain, xCT, in the brain (Sato et al., 1999; Bassi et al., 2001) — with protein expression increasing from embryonic life through adulthood (La Bella et al., 2007). In adult naïve brain, Sx_c_^–^ is expressed predominately on astrocytes (Pow, 2001; Ottestad-Hansen et al., 2018). Studies in Sx_c_^–^ null mutant mice, either in those harboring a spontaneous null mutation (SLC7A11^sut/sut^) or those resulting from conventional gene deletion of *SLC7A11* (xCT ^–/–^), have shaped our understanding of the roles this antiporter plays in central nervous system development and function.

For instance, we found that SLC7A11^sut/sut^ mice show sex-specific changes in gross, cellular, and subcellular morphology — measured at 12 weeks of age (Sears et al., 2021) — that are accompanied by alterations in excitatory/inhibitory (E/I) balance as evidenced by a decrease in convulsive seizure threshold in both male and female mice upon acute chemoconvulsant challenge (Sears et al., 2021) and a reduction in epileptogenesis (measured in male mice only) upon repeated challenge (Sears et al., 2019). Consistent with these results are the findings of enhanced hippocampal glutamatergic synaptic strength in genetically null xCT ^–/–^ male mice (Williams and Featherstone, 2014) and impairment of Schaffer collateral, CA1 synapse long term potentiation (LTP) by tetanic stimulation in SLC7A11^sut/sut^ male mice (Li et al., 2012), respectively.

Additionally, the imported cystine (CySS) from Sx_c_^–^ appears to contribute to the intracellular/extracellular redox homeostasis by facilitating the cysteine/cystine redox cycle (Banjac et al., 2008; Conrad and Sato, 2012) as well as by contributing to the synthesis of the thiol antioxidant, glutathione (GSH) (Bannai and Tateishi, 1986; Dringen, 2000). Specifically, loss of Sx_c_^–^ activity results in a redox imbalance such that xCT ^–/–^ mice [8–12 weeks; sex indeterminant] have higher CySS and lower GSH concentrations in their plasma than wildtype mice (Sato et al., 2005), although evidence of GSH reductions in cerebrum, cerebellum and hippocampus is lacking (Sato et al., 2005; De Bundel et al., 2011). In female SLC7A11^sut/sut^ mice, total levels of cysteine (CyS) and CySS did not statistically differ in the cortex, hippocampus and striatum, though a significant reduction of the CyS/CySS ratio in the cortex was found compared to age-matched SLC7A11^+/+^ littermates (Sosnoski et al., 2020). In male SLC7A11^sut/sut^ mice, a significant reduction of CyS was found in the hippocampus, but not in the cortex or striatum (Sears et al., 2019; Sosnoski et al., 2020). Total GSH levels (GSH + GSSG) were significantly higher in cortex, but not in the hippocampus or striatum in SLC7A11^sut/sut^ female mice compared to SLC7A11^+/+^; no differences were found in SLC7A11^sut/sut^ male mice. However, the ratio of reduced to oxidized GSH (GSH/GSSG) was lower in all three brain regions in female SLC7A11^sut/sut^ mice, whereas this decrease in ratio was seen only in the male striatum (Sosnoski et al., 2020).

Finally, studies in these mice provide strong evidence for the contribution of Sx_c_^–^ to the regulation of baseline glutamate concentration in the extracellular space that occurs in a sexually dimorphic and brain specific manner (De Bundel et al., 2011; Massie et al., 2011; Borra et al., 2014; McCullagh and Featherstone, 2014). Specifically, male, but not female, SLC7A11^sut/sut^ mice have been reported to have significant reductions in extracellular glutamate levels in the striatum as compared to SLC7A11^+/+^ mice, whereas the cerebellar levels of either sex were unchanged (McCullagh and Featherstone, 2014). Additionally, both young and old xCT ^–/–^ null male mice (females were not examined) showed a ≈60–65% reduction in extracellular glutamate levels in the hippocampus (De Bundel et al., 2011) and a ≈70% reduction in the striatum (age not specified) (Massie et al., 2011). So far, no study has explored glutamate levels in the cortex of these mice.

Morphological, biochemical, and electrophysiological parameters, as described above, might well be expected to alter cognitive, sensory and/or social behaviors in Sx_c_^–^ null mice. Interestingly, analysis of motor activity, motor coordination and fine motor skills in xCT ^+/+^ and xCT ^–/–^ male mice suggests that these behaviors are independent of Sx_c_^–^ function. However, adult (16–20 weeks) but not aged (19–23 months) xCT ^–/–^ mice were found to have less bright-space anxiety, whereas immobility time in the tail suspension test was reduced in xCT ^–/–^ mice of either age (Bentea et al., 2015). These results suggest that Sx_c_^–^ is dispensable for motor function but could play a potential role in anxiety and depression. Adult xCT ^–/–^ male mice have also been reported to have impairment in spatial working memory (De Bundel et al., 2011), yet hippocampus-dependent memory in xCT ^–/–^ aged male mice appears to be improved (Verbruggen et al., 2022). Previous characterization of SLC7A11^sut/sut^ mice is more limited, with one study demonstrating reduced exploratory behavior in male mice and reduced working memory in both sexes, but no deficits in motor coordination as compared to *non-littermate* wildtype control mice in either sex (McCullagh and Featherstone, 2014). Additionally, impaired fear as well as passive avoidance memory was reported in SLC7A11^sut/sut^ as compared to *non-littermate* SLC7A11^+/+^ control male mice (Li et al., 2012).

However, the full picture of the role Sx_c_^–^ plays in modulating behaviors in the SLC7A11^sut/sut^ mice is complicated by the lack of systematic and comprehensive analyses in both male and female homozygous mutant and wildtype mice derived from breeding of heterozygous parents. Thus, the aim of this study was to determine whether age- and/or sex-related changes in behavior occurs between SLC7A11^+/+^ and SLC7A11^sut/sut^ littermate mice. Toward this end, we examined the same male and female SLC7A11^+/+^ and SLC7A11^sut/sut^ mice at 2 (young) and 6 months (adult) of age using a comprehensive battery of widely employed cognitive, sensory, social and motor tests following carefully controlled breeding and housing strategies to optimally control for both genetic and environmental effects (Wolfer and Lipp, 2000; Holmdahl and Malissen, 2012) to determine if any differences in behavior due to age and/or genotype might be revealed.

## 2. Materials and methods

### 2.1. Animals and animal husbandry

The mouse line utilized in this study is on the C3H/HeSnJ background and has a large deletion mutation in Exon 12 of *SLC7A11*, which encodes for xCT, the substrate specific light chain for Sx_c_^–^ (Chintala et al., 2005). No xCT mRNA or protein is found in brain of SLC7A11^sut/sut^ mice (Chintala et al., 2005; McCullagh and Featherstone, 2014). To obtain our experimental mice, heterozygous (SLC7A11^+/sut^) breeding units were first obtained by crossing SLC7A11^sut/sut^ C3H/HeSnJ male mice [Jackson Laboratories (JAX) Stock #001310] with SLC7A11^+/+^ C3H/HeSnJ female mice (JAX, Stock #000661) or by crossing SLC7A11^sut/sut^ C3H/HeSnJ female mice [JAX, Stock #001310] with SLC7A11^+/sut^ C3H/HeSnJ male mice. Male and female F1 and F2 SLC7A11^+/sut^ progeny were then bred to obtain experimental SLC7A11*^+/+^* and SLC7A11^sut/sut^ littermates. At weaning, pups were separated two to five per cage by sex and marked for identification by ear punch. Genotyping was performed via PCR analysis of tail genomic DNA samples: +/+ primers, 5′- GAA GTG CTC CGT GAA GAA GG -3′- (forward), 5′- ATC TCA ATC CTG GGC AGA TG -3′- (reverse); sut/sut primers, 5′- CCA CTG TTG TAG GTC AGC TTA GG -3′- (forward), 5′- CAG GAC CTG TGA ATA TGA TAG GG -3′(reverse). Experimental littermate mice were then arranged such that at least one mouse of each genotype was represented in each cage. Mice were maintained in a controlled temperature environment operating on a 12 h light/dark schedule (7am/7pm) with standard mouse chow and water provided *ad libitum*. The housing and breeding strategies employed control for potential environmental differences, genetic background influences, and genetic drift (Pick and Little, 1965; Wolfer et al., 2002; Barnwell et al., 2009).

### 2.2. Behavioral tests

**FIGURE 1:**
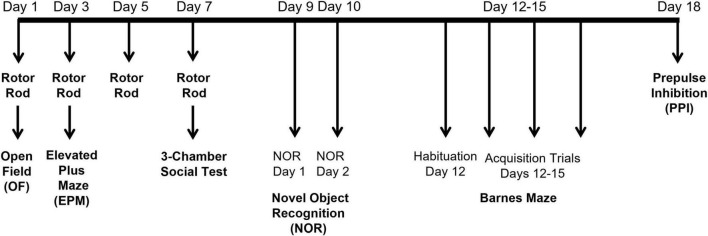
Timeline of the behavioral tests: Each mouse completed a battery of behavioral tests over 18 days in the following order: rotor rod, open field, elevated plus maze, three-chamber social interaction test, novel object recognition test, Barnes maze and pre-pulse inhibition of the acoustic startle response. The time between tests performed on the same day was at least 4 h.

Male and female SLC7A11*^+/+^* (+/+, 13M:14F) and SLC7A11^sut/sut^ (sut/sut, 15M:10F) mice at 2 (defined as young) and 6 months (defined as adult) were handled for 3 days prior to any behavioral testing. Mice were acclimated to the testing room for at least 30 min. Each apparatus was cleaned with 70% ethanol between each mouse. A battery of behavioral tests was performed over 18 days in the following order: rotor rod, open field, elevated plus maze, three-chamber social interaction test, novel object recognition test, Barnes maze and pre-pulse inhibition of the acoustic startle response (Figure 1). The time between tests performed on the same day was at least 4h. Although SLC7A11^sut/sut^ mice can initially be recognized at weaning because of their subtle gray pigmentation, we and others (Swank et al., 1996) find that this coloration becomes nearly undetectable when the mice are adults; hence, experimenters are blind to genotype at time of experimentation.

Finally, it is important to note that all C3H substrains are homozygous for the retinal degeneration 1 mutation (Pde6b^rd1^) and are blind by weaning age (Chang et al., 2002). Although some of the behavioral tests used in this study are most often used to test visuo-spatial learning and memory (i.e., Barnes maze and novel object recognition), mice with Pde6b^rd1^ mutation perform equally as well as sighted mice on both tests (O’Leary et al., 2011; Yassine et al., 2013), by relying on multisensory integration. To wit, performance of C3H substrains on the conditioned odor preference task (Brown and Wong, 2007) and their activity level paired to a 90 dB auditory cue in a fear conditioned paradigm did not differ from other sighted strains including C57BL/6J (Bolivar et al., 2001). Further, rodents are nocturnal animals, and the whisker-mediated touch system is an essential sensory system that guides them through the environment by determining objects’ shape and location [for review see Adibi (2019), Grant and Goss (2022)]. Therefore, olfaction, audition and the whisker-mediated touch system are sufficient in C3H mice to integrate the necessary information to perform the following behavioral tests.

***Rotor rod:*** The rotor rod was used to test motor performance and motor learning (Gupta et al., 2018; Eltokhi et al., 2021). Mice were placed on a still rotor rod (Ugo Basile Rotarod) for 10 sec, after which it was accelerated from 5 to 50 rpm over 5 min at a rate of 0.15 rpm/sec (3 trials per day every other day for a total of 4 testing days; intertrial interval of 10–15 min). The time to fall each day was measured. If the mouse did not fall (4 mice in total), the latency was assigned as 5 min. None of the tested mice made passive rotations [defined as one full revolution of a mouse holding onto the bar (Eltokhi et al., 2021)], as previously reported in C3H/HeSnJ mice (Gunn et al., 2011). Mice were not acclimated to the rod prior to the first trial so that we could assess motor learning, defined as difference in latency to fall at day 7 vs day 1, which is graphed as mean + SEM. Motor performance was assessed by analyzing latency to fall across days, which is graphed as the mean latency to fall ± SEM of all 3 trials. The test was conducted between 7am and 10am.

***Open Field:*** Quantitative analysis of general activity and exploration behavior was assessed in an open field using the Photobeam Activity System (PAS)-Open Field (OF) monitoring system (San Diego Instruments) with videorecording over a 30 min test period. At the start of the session, the mouse was placed in the middle of one of the side walls of the arena, facing the wall and allowed to explore. Ambulatory movement, central and peripheral activity and rearings of individual mice were measured using PAS software. The distance traveled and the percent time spent in the central zone are reported as mean + SEM by age and genotype; the number of rearings for each mouse as well as the group median is graphed by age and genotype. The test was conducted between 12pm and 6pm.

***Elevated Plus Maze:*** The elevated plus maze (EPM) was used to test for approach avoidance behavior and open-space anxiety (Walf and Frye, 2007). The mouse was placed with its head facing toward an open arm away from the experimenter after which movement was tracked and recorded by the Ethovision XT (Noldus) software for 5 min. The number of entries in the open arms for each mouse as well as the group median is graphed per age and genotype. An entry was automatically classified as the crossing of the mouse’s center point (i.e., half of the mouse body) into the open arms. The percent time spent in the open arms [(time in open arms/time in all arms) x 100] was calculated for each mouse and is graphed as mean + SEM by age and genotype. The test was conducted between 12pm and 4pm.

***Olfaction Test:*** To confirm olfaction in C3H/HeSnJ mice, odor-induced c-fos mRNA levels in olfactory bulbs of mice at 2 months of age was measured (Byrne et al., 2022). Each mouse was placed in an empty clean holding cage containing an unscented cotton swab suspended from the wire lid. After 2 h, the unscented cotton swab was replaced with either a novel unscented (control) cotton swab (+ / + , *n* = 4M:6F; sut/sut, *n* = 5M:6F) or a cotton swab saturated with a commercially available peppermint extract (+ / + , *n* = 4M:6F; sut/sut, *n* = 4M:6F). After 30 min, the mice were euthanized and olfactory bulbs were dissected and placed in TRIzol reagent (Invitrogen, Carsbad, CA). Samples were stored at −20°C overnight. Total RNA was isolated and first-strand complementary DNA (cDNA) synthesized as described (Kanzler et al., 2018). cDNA samples were amplified for 40 cycles (95°C for 15sec, 60°C for 60sec, 95°C for 15 sec, 60°C for 15 sec and 95°C for 50 sec) using Taq DNA polymerase (Invitrogen) and target specific mouse primers for c-fos (Mm00487425_m1, TaqMan Gene Expression Assays, Applied Biosystems, Foster City, CA) and hypoxanthine guanine phosphoribosyl transferase (HPRT, Mm01545399_m1, TaqMan Gene Expression Assays, Applied Biosystems). Reactions were performed in triplicate using a BIO-RAD CFX Connect Real Time System. Data analysis was performed using the comparative cycle threshold method (2^–ΔΔ^C_T_), where C_T_ values of c-fos were normalized to HPRT C_T_ values from the same sample, then compared to calibrator C_T_ values (non-odor control within genotype) to determine the relative fold increase in c-fos mRNA. Sample specific PCR efficiencies were performed and did not differ by more than 5% between primers. HPRT C_T_ values were unaffected by the experimental conditions.

***3-Chamber Social Test:*** The 3-chamber test was used to measure social interaction (Nadler et al., 2004). The mouse was initially placed in the middle chamber with dividers closed for 10 min. Subsequently, the dividers were removed, allowing the mouse to freely explore all three vacant chambers for 10 min. Following these habituation periods, the mouse was constrained in the middle chamber while an empty wire cage was placed in one lateral chamber, and an identical wire cage containing a stranger mouse was placed in the other (alternating placement between each mouse tested). The sex- and age-matched, non-littermate stranger mouse was habituated to the wire cup for two 15 min sessions prior to the experiment. The dividers were raised, and the time spent in each chamber and time spent within 2 cm (whisker length 2.5 ± 0.04 cm; *n* = 10M:8F) of each cage was automatically recorded by the Ethovision XT software system (Noldus). A total of 11 mice (+ / + , *n* = 2M:4F; sut/sut, *n* = 3M:2F) in the young group and 8 mice (+ / + , *n* = 1M:5F; sut/sut, *n* = 2M) in the adult group were excluded due to side preference — defined as spending more than 60% of the total time or less than 10 sec time in one of the chambers during the habituation period to the three chambers. Time spent on top of the wire cages was not counted as interaction time and was subtracted from the total interaction time automatically recorded. Data are graphed as mean time (sec) by age and genotype. The test was conducted between 12pm and 6pm.

***Novel Object Recognition:*** The Novel Object Recognition (NOR) is used to quantify recognition memory (Ennaceur and Delacour, 1988). We used objects shaped as pyramids and cylinders to enable whisker-based shape discrimination (Rodgers et al., 2021). The mouse was placed in a square empty chamber for 10 min to habituate, after which the mouse was removed and two identical objects were placed in the chamber on opposite sides, 17 cm apart. The mouse was returned to the chamber and was allowed to explore the two identical objects for 10 min — the familiarization session. Twenty-four h later, the chamber was set with one familiar object (same shape as the previous day) and one novel object (different shape), then the mouse was placed in the middle of the chamber and allow to explore for 10 min — the testing session. Mouse movements were recorded using Ethovision XT software (Noldus). Two experimenters blind to the genotype of the mouse recorded the amount of time its nose was sniffing or touching the objects. Sitting on the object was not counted as exploration time (Ennaceur and Delacour, 1988; de Lima et al., 2011). If there was a disagreement a third experimenter, also blind to genotype, scored the trial. The average of the two scores were taken as the exploration time for each object after the rating performance of the experimenters was compared using Pearson’s correlation analysis on the % time exploring the novel object — calculated as follows [time novel object/(time novel object + time familiar object)] — between each observer (Shapiro Wilk test *p* > 0.05; 0.85 < r < 0.99, *p* ≤ 0.0002 Pearson’s Correlation) (Benice and Raber, 2008). Data are graphed as mean exploration time (sec) + SEM. The discrimination index was calculated as the difference in time exploring the new object and the familiar object over the total time exploring both objects and graphed as mean + SEM. If a mouse spent less than 20 sec exploring both objects (i.e., total exploration time) during the 10 min familiarization or testing session it was excluded (sut/sut, *n* = 1M, young). The test was conducted between 12pm and 6pm.

***Barnes Maze:*** The Barnes Maze (Barnes, 1979) — an elevated circular platform with 20-open holes at the periphery — was used to test spatial learning and memory. Prior to the first training day, mice were habituated to the escape chamber (2 min), whereafter they were placed in the middle of the platform sans escape chamber to allow for 2 min of exploration. Immediately following habituation, a stair built with LEGO^®^ blocks was placed in the escape chamber to facilitate the mouse’s entrance by whisker-mediated touch. The position of the escape chamber was kept constant during the acquisition trials for each mouse; however, it varied between mice. The first acquisition trial began by placing the mouse in the middle of the maze. Each mouse completed 3 trials per day/4 consecutive days total (3 min per trial; intertrial period of at least 30 min). On each day, the primary latency to the escape hole (i.e., the first contact, nose poke and/or the front paws placed into the hole with the escape chamber) was recorded and expressed as mean time to hole (sec) ± SEM by age and genotype for each day of training. Additionally, the search strategy employed (direct, serial, or random) was recorded (Gawel et al., 2019). A mouse utilizes a direct strategy if it moves immediately to the escape chamber or visits 1–2 hole(s) adjacent before entering the escape chamber. If the first visit to the escape chamber is preceded by visitation of two holes adjacent to each other but not to the escape chamber, a serial strategy is said to be employed. An unorganized search, characterized by crossing through the center of the maze, is defined as random. The strategies used per day were calculated as follows: [(frequency of each search strategy employed per day/total # of trails per day) × 100] and graphed. Mouse movements was recorded by Ethovision XT (Noldus). A total of 3 mice (+ / + , *n* = 2M: 1F) in the young group, and 2 mice (+ / + , *n* = 1F; sut/sut, *n* = 1M) in the adult group were excluded due to experimental error. Test was performed between 10am and 6pm.

***Acoustic Startle Response, Prepulse Inhibition, Habituation:*** Prepulse inhibition of the acoustic startle response is used to assess sensorimotor gating (Johansson et al., 1995). The mouse was placed in a SR-LAB startle chamber (San Diego Instruments) for a 3 min acclimation period prior to the delivery of any stimulus. The first and last 6 trials consisted of the startle stimulus alone (120 dB, 40 ms). Remaining trials occurred in a pseudo-random order: 12 stimuli at each of the 4 prepulse intensities [6, 12, 15, 18 dB above 70 dB background (20 ms)] preceded the startle stimulus by 100 ms; 8 no stimulus trials; and 12 startle trials without a prepulse stimulus. The inter-trial interval averaged 15 sec. Startle reactivity is graphed as the mean ± SEM of the 12 pseudo-random startle trials and of the 4 prepulse intensities. The percent PPI is graphed as mean + SEM PPI [100 – (100 × [mean prepulse/mean startle])]. Habituation is graphed as a percentage and was calculated as follows: (100 × [(mean startle amplitude on the first 6 trials – mean startle amplitude on the last 6 trials)/mean startle amplitude on the first 6 trials]). Hence, if there is no change in magnitude of the startle response from the beginning to end of the paradigm, the habituation percentage will be 0%. A positive habituation percentage indicates mice have habituated to the stimulus, whereas a negative habituation percentage indicates sensitization to the stimulus. Test was performed between 10am and 6pm.

### 2.3. Statistical analysis

All statistical analyses were performed using GraphPad Prism (Version 9.2.0 Graphpad Software, Inc., La Jolla, CA or higher). Prior to parametric analysis, data were tested for normality using the Shapiro-Wilk test; non-normal data were transformed using the Y squared or log(Y) functions in GraphPad Prism. Percentage data or counted data, which are by nature non-normal, were transformed via arcsine transformation (y = arcsine[sqrt(y)]) and via sqrt(Y) transformation, respectively. When possible, a non-parametric test was employed. Two-way ANOVA followed by Bonferroni’s multiple comparisons test were performed to analyze the data by genotype at each age and mixed model repeated measures (MMRM) ANOVA followed by Bonferroni’s multiple comparisons test was performed to analyze the data by age for each genotype. When comparing differences between the first and the last day of training, Dunnett’s multiple comparisons test was used. As percent habituation was not normally distributed and negative values could not be transformed via arcsine transformation, we performed RM ANOVA on the habituation score defined as [(mean startle amplitude on the last 6 trials)/(mean startle amplitude on the first 6 trials)]. To enhance readability, exact p values and tests are only described in the text if the results are unaccompanied by a figure, else they can be found in the figure legends.

## 3. Results

### 3.1. Body weight

Both male and female SLC7A11*^+/+^* and SLC7A11^sut/sut^ mice increased their body weight over the testing period by 20–24% with no differences between genotypes found by MMRM ANOVA [F_(1_,_26)_ = 1.950, *p* = 0.1744, genotype males; F_(1_,_22)_ = 0.3116, *p* = 0.5824 genotype females]. Male SLC7A11*^+/+^* weighed an average of 27.1 ± 1.1 g at 2 months increasing to 35.4 ± 1.2 g by 6 months of age, whereas male SLC7A11^sut/sut^ weighed on average 25.8 ± 0.7 g at 2 months increasing to 32.7 ± 1.1 g by 6 months of age. The average body weight of female SLC7A11*^+/+^* and SLC7A11^sut/sut^ was 22.1 ± 0.6 g and 23.3 ± 0.9 g, respectively, increasing to 29.2 ± 0.8 g and 29.2 ± 0.9 g, respectively. Therefore, body weight does not contribute to genotype behavioral differences found in this study.

### 3.2. Rotor rod test

**FIGURE 2:**
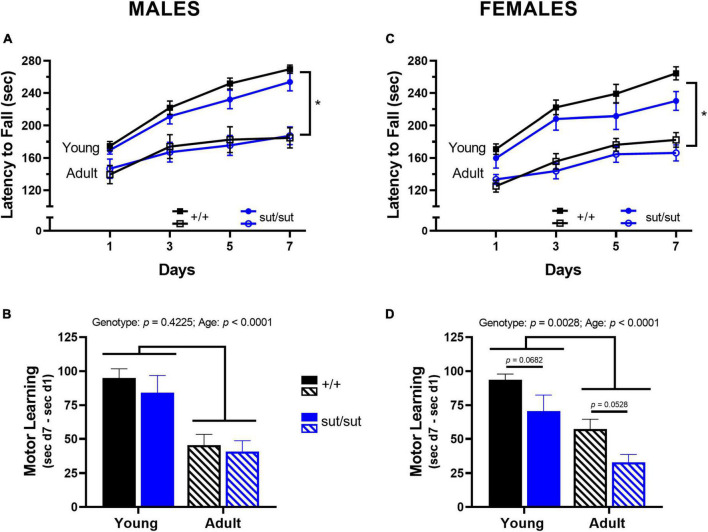
Rotor rod performance. Male **(A,B)** or Female **(C,D)** SLC7A11^+/+^ (+ / + , *n* = 13M:14F, black) and SLC7A11^sut/sut^ (sut/sut, *n* = 15M:10F, blue) littermates were tested on the accelerating rotor rod at 2 (young) or 6 months (adult). Motor performance is measured as latency to fall across days and plotted as mean ± SEM. Motor learning is defined as difference in latency to fall at day 7 (d7) vs day 1 (d1) and plotted as mean + SEM. All data were analyzed via MMRM ANOVA followed by Bonferroni’s multiple comparisons test. No within genotype sex differences were found. **(A)** Performance in males increased across the testing days but did not differ between genotypes at either age: *Young:* F_(2_._174_, _56_._51)_ = 75.87, *p* < 0.0001, testing days; F_(1_, _26)_ = 1.921, *p* = 0.1775, genotype; F_(3_, _78)_ = 0.5327, *p* = 0.6612, interaction; *Adult:* F_(2_._200_, _57_._19)_ = 23.75, *p* < 0.0001, testing days; F_(1_, _26)_ = 0.0037, *p* = 0.9518, genotype; F_(3_, _78)_ = 0.8511, *p* = 0.4702, interaction. *Age negatively affected performance in both genotypes +/+ : F_(1_, _12)_ = 27.49, *p* = 0.0002 for age; sut/sut: F_(1_,_14)_ = 16.75, *p* = 0.0011 for age. **(B)** Motor learning in males was negatively affected by age independent of genotype: F_(1_,_26)_ = 0.6641, *p* = 0.4225, genotype; F_(1_, _26)_ = 25.50, *p* < 0.0001, age; F_(1_,_26)_ = 0.1138, *p* = 0.7385, interaction (young vs adult: *p* = 0.0021 for +/+ , *p* = 0.0038, for sut/sut) (+ / + vs sut/sut: *p* = 0.8298 for young; *p* > 0.9999 for adult). **(C)** Performance in females increased across the testing days but did not differ between genotypes at either age: *Young*: F_(2_._633_, _57_._92)_ = 49.50, *p* < 0.0001, testing days; F_(1_, _22)_ = 2.689, *p* = 0.1153, genotype; F_(3_, _66)_ = 1.241, *p* = 0.3018, interaction; *Adult*: F_(2_._709_, _59_._60)_ = 37.16, *p* < 0.0001, testing days; F_(1_, _22)_ = 0.4923, *p* = 0.4902, genotype; F_(3_, _66)_ = 2.571, *p* = 0.0616, interaction. *Age negatively affected performance of mice of both genotypes: +/+ : F_(1_, _15)_ = 50.80, p < 0.0001 for age; sut/sut: F_(1_,_11)_ = 13.57, *p* = 0.0036 for age. **(D)** Motor learning in females was negatively affected by age in both genotypes: F_(1_,_44)_ = 10.06, *p* = 0.0028, genotype; F_(1_, _44)_ = 24.48, *p* < 0.0001, age; F_(1_,_44)_ = 0.0061, *p* = 0.9379, interaction (young vs adult: *p* = 0.0010 for +/+ ; *p* = 0.0040 for sut/sut) with additional impairment in learning evident in both young and adult sut/sut mice as compared to their age-matched +/+ littermates (+ / + vs sut/sut: *p* = 0.0682 for young, *p* = 0.0528 for adult).

Repeated experience on the rotor rod increased motor performance in male mice in a genotype-independent manner at both 2 months and 6 months, despite the overall performance of both genotypes being negatively affected by age (Figure 2A). Motor learning in male mice — defined as difference in latency to fall at day 7 vs day 1 — was also negatively affected by age in a genotype-independent manner (Figure 2B). Likewise, in female mice, a genotype-independent increase in intersession performance was found at both ages. As with males, age negatively affected performance (Figure 2C) and motor learning (Figure 2D) of female mice of both genotypes. However, female SLC7A11^sut/sut^ mice showed an additional impairment in learning as compared to their SLC7A11*^+/+^* littermates at both ages (Figure 2D). Hence, it appears that functional Sx_c_^–^ signaling is important for motor learning in females only.

### 3.3. Open field test

**FIGURE 3:**
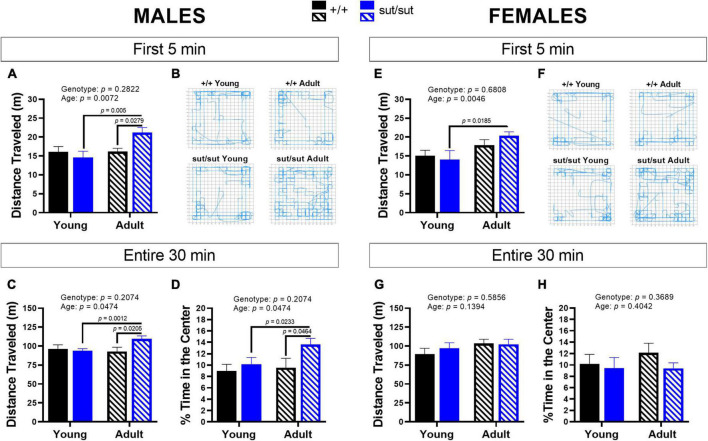
Open field activity. Male **(A–D)** or Female **(E–H)** SLC7A11^+/+^ (+ / + , n = 13M:14F, black bars) and SLC7A11^sut/sut^ (sut/sut, n = 15M:10F, blue bars) littermates were tested at 2 (young) or 6 months (adult). Ambulatory movement (distance traveled), central and peripheral activity (% time in center) of individual mice were measured. Data are plotted as mean + SEM and analyzed using MMRM ANOVA followed by the Bonferroni’s multiple comparisons test. No within genotype sex differences were found. **(A)** *Distance traveled by males (5 min):* F_(1_,_26)_ = 8.520, *p* = 0.0072, age; F_(1_,_26)_ = 1.206, *p* = 0.2822, genotype; F_(1_,_26)_ = 8.078, *p* = 0.0086, interaction. (+ / + vs sut/sut: *p* = 0.9056 for young; *p* = 0.0279 for adult) (young vs adult: *p* = 0.0005 for sut/sut; *p* > 0.9999 for + / +). **(B)** *Paths.* Representative activity in of the first 5 min in the open filed by the same male mice at 2 (young) and 6 months (old). **(C)** *Distance traveled by males (30 min):* F_(1_, _26)_ = 4.333, *p* = 0.0474 age; F_(1_, _26)_ = 1.672, *p* = 0.2074, genotype; F_(1_, _26)_ = 10.4, *p* = 0.0034, interaction. (+ / + vs sut/sut: *p* > 0.9999 for young; *p* = 0.0205 for adult) (young vs adult: *p* = 0.0012 for sut/sut; *p* = 0.8836 for + / +). **(D)%** *time in the center (30 min) – males:* F_(1_, _26)_ = 3.513, *p* = 0.0722, age; F_(1_, _26)_ = 2.837 *p* = 0.1041, genotype; F_(2_,_26)_ = 3.330, *p* = 0.0796, interaction. (+ / + vs sut/sut: *p* > 0.9999 for young; *p* = 0.0464 for adult) (young vs adult: *p* = 0.0233 for sut/sut; *p* > 0.9999 for + / +). **(E)** *Distance traveled by females (5 min):* F_(1_,_22)_ = 9.944, *p* = 0.0046, age; F_(1_,_22)_ = 0.1738, *p* = 0.6808, genotype; F_(1_,_22)_ = 1.450, *p* = 0.2412, interaction. (+ / + vs sut/sut: *p* > 0.9999 for young; *p* = 0.5801 for adult) (young vs adult: *p* = 0.0185, for sut/sut; *p* = 0.2907 for + / +). **(F)** *Paths.* Representative activity in of the first 5 min in the open filed by the same female mice at 2 (young) and 6 months (old). **(G)** *Distance traveled by females (30 min):* F_(1_,_22)_ = 2.352, *p* = 0.1394, age; F_(1_,_22)_ = 0.3062, *p* = 0.5856, genotype; F_(1_,_22)_ = 0.7756, *p* = 0.3880, interaction. (+ / + vs sut/sut: *p* = 0.6685 for young; *p* > 0.9999 for adult) (young vs adult: *p* > 0.9999 for sut/sut; *p* = 0.1497 for + / +). **(H)**% *time in the center (30 min) – females:* F_(1_, _22)_ = 0.7235, *p* = 0.4042, age; F_(1_, _22)_ = 0.8414, *p* = 0.3689, genotype; F_(2_, _22)_ = 0.3583, *p* = 0.5555, interaction. (+ / + vs sut/sut: *p* > 0.9999 for young; *p* = 0.5748 for adult) (young vs adult: *p* > 0.9999 for sut/sut; *p* = 0.5475 for + / +).

**FIGURE 4:**
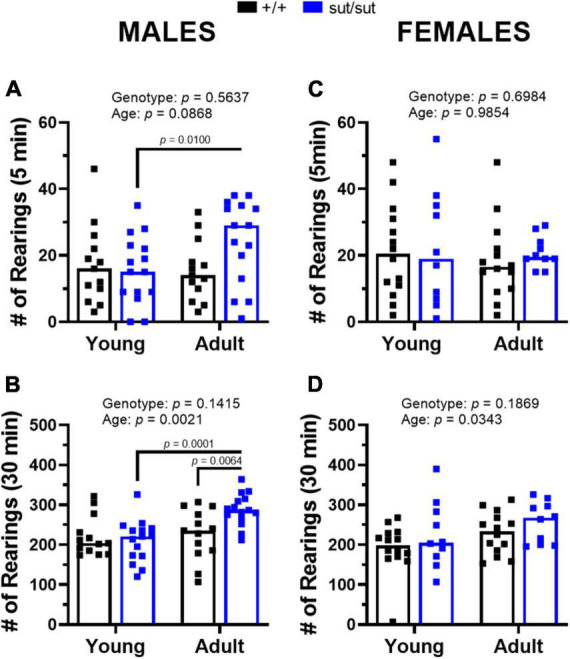
Rearings behavior in the open field. Male **(A,B)** or Female **(C,D)** SLC7A11^+/+^ (+ / + , n = 13M:14F, black bars) and SLC7A11^sut/sut^ (sut/sut, n = 15M:10F, blue bars) littermates were tested at 2 (young) or 6 months (adult). Each data point representing an individual animal is graphed with the median number of rearings reflected by horizontal line of the bar. Following transformation, data were analyzed using MMRM ANOVA followed by the Bonferroni’s multiple comparisons test. No within genotype sex differences were found. **(A)** *Male rearings (5 min)*: F_(1_,_26)_ = 3.167, *p* = 0.0868, age; F_(1_,_26)_ = 0.3420, *p* = 0.5637, genotype; F_(1_,_26)_ = 5.770, *p* = 0.0237, interaction. (+ / + vs sut/sut: *p* > 0.9999 for young; *p* = 0.2235 for adult) (young vs adult: *p* = 0.0100 for sut/sut; *p* > 0.9999 for + / +). **(B)** *Male rearings (30 min):* F_(1_,_26)_ = 11.67, *p* = 0.0021, age; F_(1_,_26)_ = 2.299, *p* = 0.1415, genotype; F_(1_,_26)_ = 10.15, *p* = 0.0037, interaction. (+ / + vs sut/sut: *p* > 0.9999 for young; *p* = 0.0064 for adult) (young vs adult: *p* = 0.0001 for sut/sut; *p* > 0.9999 for + / +). **(C)** *Female rearings (5 min):* F_(1_,_22)_ = 0.0003, *p* = 0.9854, age; F_(1_,_22)_ = 0.1541, *p* = 0.6984, genotype; F_(1_,_22)_ = 0.6836, *p* = 0.4172, interaction. (+ / + vs sut/sut: *p* > 0.9999 for young; *p* = 0.8850 for adult) (young vs adult: *p* > 0.9999 for sut/sut; *p* > 0.9999 for + / +). **(D)** *Females rearings (30 min):* F_(1_,_22)_ = 5.094 *p* = 0.0343, age; F_(1_,_22)_ = 1.856, *p* = 0.1869, genotype; F_(1_,_22)_ = 0.1047, *p* = 0.7494, interaction. (+ / + vs sut/sut: *p* = 0.4098 for young; *p* = 0.7448 for adult) (young vs adult: *p* = 0.4376 for sut/sut; *p* = 0.1162 for + / +).

Distanced traveled by male mice revealed a main effect of age and a significant age x genotype interaction during the first 5 min as well as throughout the 30 min session (Figure 3). *Post hoc* analyses demonstrated enhanced locomotor activity with age in male SLC7A11^sut/sut^ at both 5 min and 30 min. Adult male SLC7A11^sut/sut^ mice also traveled greater distances than age matched SLC7A11*^+/+^* mice at both 5 and 30 min (Figures 3A–C). Adult SLC7A11^sut/sut^ male mice also spent more time in the center of the open field as compared to both young SLC7A11^sut/sut^ and adult SLC7A11*^+/+^* male mice (Figure 3D). Finally, adult male SLC7A11^sut/sut^ also have increased rearing behavior as compared to young male SLC7A11^sut/sut^ mice at 5 min and as compared to both young SLC7A11^sut/sut^ and adult SLC7A11*^+/+^* mice at 30 min (Figures 4A, B). All together these results suggest that behavioral hyperactivity occurs with age in SLC7A11^sut/sut^ male mice.

With respect to females, exploratory behavior in the adult SLC7A11^sut/sut^ mice increased in the first 5 min as compared to the young SLC7A11^sut/sut^ females but, unlike with males, this increase did not persist throughout the 30 min session (Figures 3E–G). Additionally, female mice did not differ by age or genotype in the percent time spent in the center (Figure 3H). Finally, the number of rearings in female SLC7A11^sut/sut^ mice also did not differ by age or genotype in either the first 5 min or the entire 30 min of the paradigm, even though a main effect of age was present (Figures 4C, D). Hence, overall SLC7A11^sut/sut^ female mice do not display a similar hyperactivity phenotype as seen with males.

### 3.4. Elevated plus maze test

**FIGURE 5:**
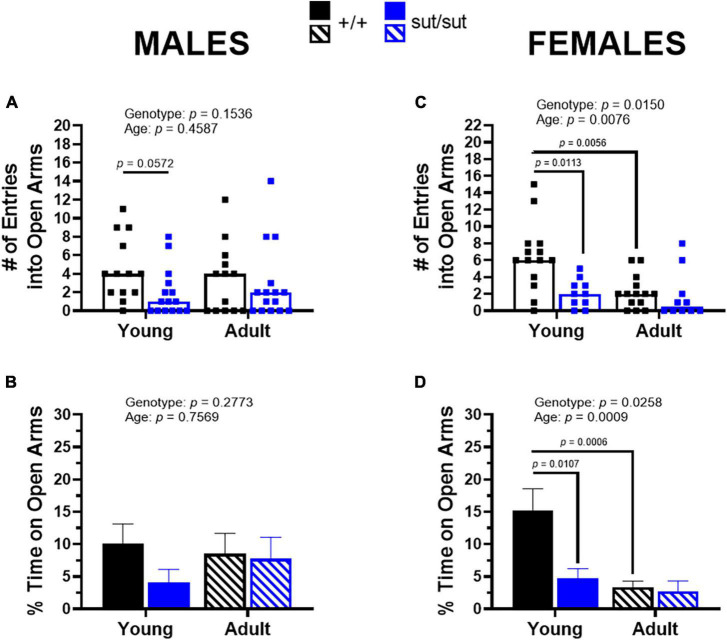
Elevated plus maze. Male **(A,B)** or Female **(C,D)** SLC7A11*^+/+^* (+ / + , n = 13M:14F, black) and SLC7A11*^sut/sut^* (sut/sut, n = 15M:10F, blue) littermates were tested at 2 (young) or 6 months (adult). Each data point representing an individual animal is graphed with the median number of entries reflected by horizontal line of the bar. The% time spent on the open arms is graphed as mean + SEM. Following transformation, data were analyzed via MMRM ANOVA followed by Bonferroni’s multiple comparisons test. No within genotype sex differences were found. **(A)** *Number of entries — males:* F_(1_, _26)_ = 2.160, *p* = 0.1536, genotype; F_(1_, _26)_ = 0.5658, *p* = 0.4587, age; F_(1_, _26)_ = 3.933, *p* = 0.0580, interaction. (+ / + vs sut/sut: p = 0.0572 for young; *p* > 0.9999 for adult) (young vs adult: *p* = 0.1460 for +/+ ; *p* = 0.7493 for sut/sut). **(B)**% *time on open arms — males:* F_(1_, _26)_ = 1.231, *p* = 0.2773, genotype; F_(1_, _26)_ = 0.09789, *p* = 0.7569, age; F_(1_, _26)_ = 3.514, *p* = 0.0721, interaction. (+ / + vs sut/sut: *p* = 0.1489 for young; *p* > 0.9999 for adult) (young vs adult: *p* = 0.5917 for +/+ ; *p* = 0.2411 for sut/sut). **(C)** *Number of entries — females:* F_(1_, _22)_ = 6.967, *p* = 0.0150, genotype; F_(1_, _22)_ = 8.620, *p* = 0.0076, age; F_(1_, _22)_ = 1.979, *p* = 0.1734, interaction. (+ / + vs sut/sut: *p* = 0.0113 for young; *p* = 0.6044 for adult) (young vs adult: *p* = 0.0056 for +/+ ; *p* = 0.6554 for sut/sut). **(D)**% *time on open arms — females:* F_(1_, _22)_ = 5.716, *p* = 0.0258 genotype; F_(1_, _22)_ = 14.81. *p* = 0.0009, age; F_(1_, _22)_ = 2.891, *p* = 0.1031, interaction. (+ / + vs sut/sut: *p* = 0.0107 for young; *p* = 0.8191 for adult) (young vs adult: *p* = 0.0006 for +/+ ; *p* = 0.3473 for sut/sut).

Analysis of behavior in the EPM demonstrated that both male and female SLC7A11^sut/sut^ mice show increased open-space anxiety compared to male and female SLC7A11*^+/+^* mice. This was evidenced by a reduction in entries into the open arms during the 5 min testing period. Specifically, median entries of young male and female SLC7A11*^+/+^* mice equal 4 and 6, respectively, whereas median entries for young male and female SLC7A11^sut/sut^ mice are just 1 and 2, respectively (Figures 5A, C). This corresponds to a reduction in the percent time spent in the open arms by young male and female SLC7A11^sut/sut^ mice as compared to their age-matched SLC7A11*^+/+^* littermate controls, although this was only statistically significant in the females (Figures 5B, D). These data demonstrate that overall, young SLC7A11^sut/sut^ mice appear to show enhanced open-space anxiety when compared to their littermate controls. In male and female adult SLC7A11^sut/sut^ mice, open arm entries are reduced by 50 and 75%, respectively, when compared to their age- and sex-matched SLC7A11*^+/+^* mice; however, these differences were not statistically significant (Figures 5A, C). The percent time in the open arms also did not statistically differ between genotype in adult male and female mice (Figures 5B, D).

Finally, age significantly worsened open-space anxiety in SLC7A11*^+/+^* female mice — but not male SLC7A11*^+/+^* mice — as demonstrated by significant reduction in the number of entries into open arms and the percent time spent in the open arms when compared to when they were young (Figures 5C, D). Similar to findings in male SLC7A11*^+/+^* mice, age did not enhance open-space anxiety in either male or female SLC7A11^sut/sut^ mice. On the contrary, in males the median number of entries and the percent time in the center was higher as compared to when they were younger (Figures 5A, B). This may be attributable to the age-dependent increase in exploratory behavior that was uncovered in male SLC7A11^sut/sut^ mice in the open field.

### 3.5. 3-chamber social test

*Olfaction.* Prior to analyzing sociability, we confirmed intact olfaction by quantifying c-fos mRNA expression in the olfactory bulbs in response to novel odorant exposure. An odor-induced increase in c-fos mRNA was measured in all mice regardless of genotype (Supplementary Figure 1). Hence, olfaction is intact in our mice.

**FIGURE 6:**
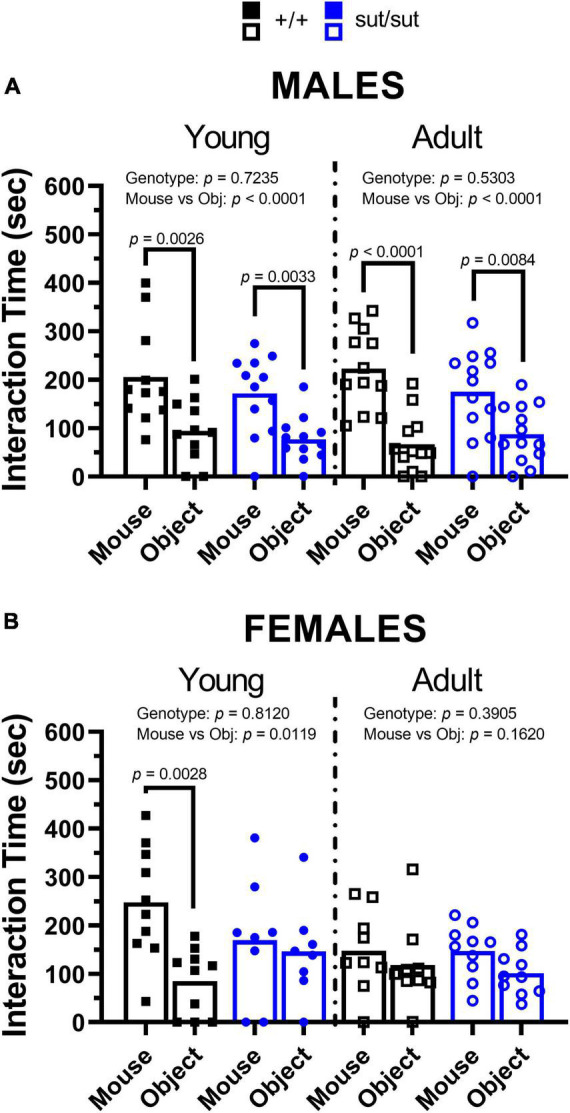
3-chamber social test. Male **(A)** or Female **(B)** SLC7A11*^+/+^* and SLC7A11*^sut/sut^* littermates were tested at 2 (young) [ +/+ , n = 11M:10F, black; sut/sut, n = 12M:8F, blue] and at 6 months (adult) [ +/+ , n = 13M:9F, black; sut/sut, n = 13M:10F, blue]. Each data point representing an individual animal is graphed with the mean interaction time reflected by horizontal line of the bar. Data were analyzed within each age group by two-way ANOVA followed by Bonferroni’s multiple comparisons test. **(A)** *Time interacting with mouse vs object in males: Young*: F_(1_, _42)_ = 20.78, *p* < 0.0001, mouse/object; F_(1_, _42)_ = 1.247, *p* = 0.2705, genotype; F_(1_, _42)_ = 0.1268, *p* = 0.7235, interaction. (mouse vs object: *p* = 0.0026 for +/+ , *p* = 0.0033 for sut/sut). *Adult*: F_(1_, _46)_ = 33.63, *p* < 0.0001, mouse/object; F_(1_, _46)_ = 0.3998, *p* = 0.5303, genotype; F_(1_, _46)_ = 2.644, *p* = 0.1108, interaction. (mouse vs object: *p* < 0.0001 for +/+ , *p* = 0.0084 for sut/sut). **(B)** *Time interacting with mouse vs object in females: Young*: F_(1_, _32)_ = 7.113, *p* = 0.0119, mouse/object; F_(1_, _32)_ = 0.0575, *p* = 0.8120, genotype; F_(1_, _32)_ = 3.995, *p* = 0.0542, interaction. (mouse vs object: *p* = 0.0028 for +/+ , *p* > 0.9999 for sut/sut). *Adult:* F_(1_, _34)_ = 2.043, *p* = 0.1620, mouse/object; F_(1_, _34)_ = 0.7565, *p* = 0.3905, genotype; F_(1_, _34)_ = 0.1070, *p* = 0.7456, interaction. (mouse vs object: *p* = 0.9054 for +/+ , *p* = 0.4212 for sut/sut).

*Sociability.* No socialization deficits were found in male mice regardless of genotype or age (Figure 6A). Male mouse regardless of genotype also preferred the mouse chamber over that containing the object, as might be expected, although significant differences were noted for SLC7A11*^+/+^* male mice only (Supplementary Figure 2A). Exploratory behavior, measured as number of entries in each chamber, was similar between genotypes at 2 and 6 months (Supplementary Figure 2C).

In contrast, social behavior was negatively affected by loss of Sx_c_^–^ in young females. The deficit found in female SLC7A11^sut/sut^ mice persisted into adulthood and strikingly emerged in the SLC7A11*^+/+^* female mice (Figure 6B). The time spent within the two chambers containing the mouse or object, which did not differ, mirrors precisely the direct interaction time at each age (Supplementary Figure 2B) and exploratory behavior was similar between genotypes (Supplementary Figure 2D). Altogether, these results emphasize age and sex differences in social behavior with Sx_c_^–^ contributing to normal social behavior in females, but not males, and with age impacting sociability in female SLC7A11*^+/+^* mice only.

### 3.6. Novel object recognition

**FIGURE 7:**
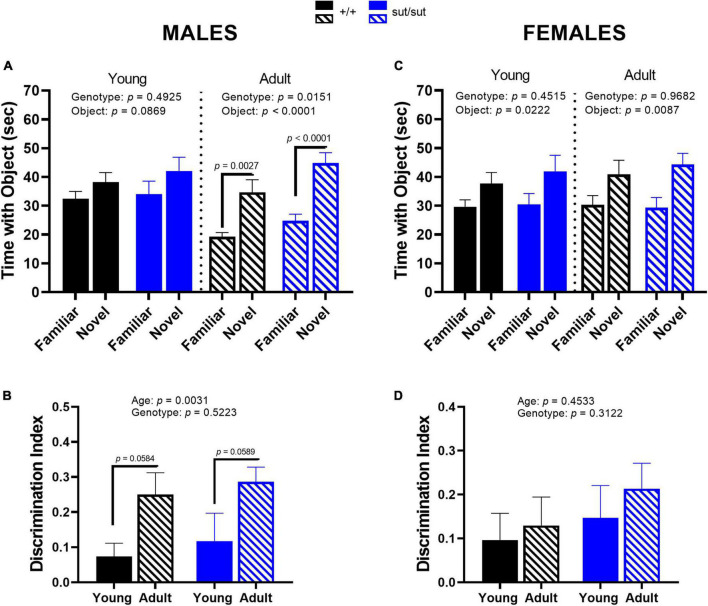
Novel object recognition. Male **(A,B)** or Female **(C,D)** SLC7A11^+/+^ and SLC7A11^sut/sut^ littermates were tested at 2 (young) [ +/+ , n = 13M:14F, black; sut/sut, n = 14M:10F, blue] and at 6 months (adult) [ +/+ , n = 13M:14F, hatch black; sut/sut, n = 15M:10F, hatch blue]. The mean time + SEM interacting with the novel and familiar object is graphed and analyzed within each age group by two-way ANOVA followed by Bonferroni’s multiple comparisons test. The discrimination index, calculated as the difference in time exploring the new object and the familiar object over the total time exploring both objects, was graphed as mean + SEM and analyzed via MMRM ANOVA followed by Bonferroni’s multiple comparisons test. **(A)** *Time interacting with mouse vs object in males: Young*: F_(1_,_50)_ = 3.051, *p* = 0.0869, object; F_(1_,_50)_ = 0.4780, *p* = 0.4925, genotype; F_(1_,_50)_ = 0.0801, *p* = 0.7784, interaction (novel vs familiar: *p* = 0.6288 for +/+ , *p* = 0.2998 for sut/sut). *Adult*: F_(1_,_52)_ = 32.32, *p* < 0.0001, object; F_(1_,_52)_ = 6.316, *p* = 0.0151, genotype; F_(1_,_52)_ = 0.5293, *p* = 0.4701, interaction. (novel vs familiar: *p* = 0.0027 for +/+ , *p* < 0.0001 for sut/sut). **(B)** *Discrimination index in male mice*: F_(1_, _25)_ = 10.68, *p* = 0.0031, age; F_(1_, _26)_ = 0.4207, *p* = 0.5223, genotype; F_(1_, _25)_ = 0.0074, *p* = 0.9318, interaction. (adult vs young: *p* = 0.0584 for +/+ , *p* = 0.0589 for sut/sut). **(C)** *Time interacting with mouse vs object in females: Young:* F_(1_,_44)_ = 0.5771, *p* = 0.4515, genotype; F_(1_,_44)_ = 5.620, *p* = 0.0222, object; F_(1_,_44)_ = 0.2122, *p* = 0.6473, interaction. (novel vs familiar: *p* = 0.2923 for +/+ , *p* = 0.1410 for sut/sut). *Adult:* F_(1_,_44)_ = 0.0016, *p* = 0.9682, genotype; F_(1_,_44)_ = 7.543, *p* = 0.0087, object; F_(1_,_44)_ = 0.0657, *p* = 0.7988, interaction. (novel vs familiar: *p* = 0.1204 for +/+ , *p* = 0.1113 for sut/sut). **(D)** *Discrimination index in female mice*: F_(1_, _44)_ = 0.5724, *p* = 0.4533, age; F_(1_, _44)_ = 1.045, *p* = 0.3122, genotype; F_(1_, _44)_ = 0.06248, *p* = 0.8023 interaction (adult vs young: *p* > 0.9999 for both +/+ and sut/sut).

Recognition memory in male mice improved with age, regardless of genotype, as adult mice spent significantly more time exploring the novel object as compared to the familiar object than when they were younger (Figure 7A). This enhancement is further demonstrated by an increase in the discrimination index in adult mice (Figure 7B). Female mice showed an overall genotype-independent preference for the novel object at both ages tested, although no significant within-group differences were found on *post hoc* analyses (Figure 7C). Additionally, the discrimination index in females did not differ by age or genotype (Figure 7D). Altogether, these results indicate that Sx_c_^–^ signaling is not necessary for recognition memory in mice of either sex. However, age and sex do effect recognition memory: young and adult female mice show similar innate preference in investigating the novel object, while this preference is enhanced with age in male mice.

### 3.7. Barnes maze

**FIGURE 8:**
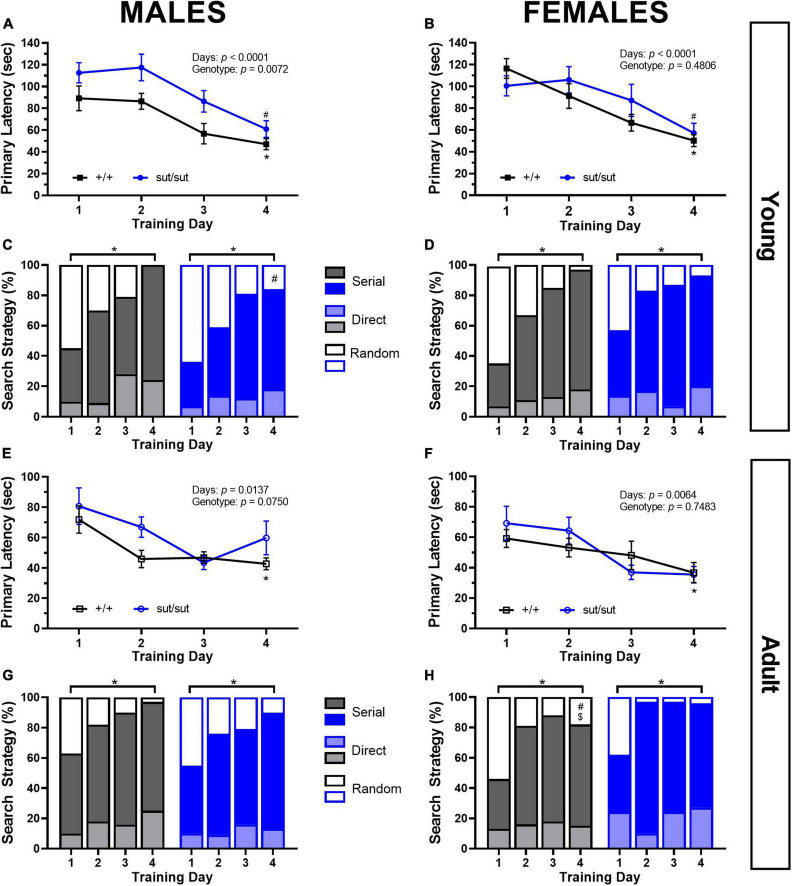
Barnes maze performance. Male or Female SLC7A11*^+/+^* and SLC7A11*^sut/sut^* littermates were tested at 2 (young) [ +/+ , *n* = 11M:13F, black; sut/sut, *n* = 15M:10F, blue] and at 6 months (adult) [ +/+ , *n* = 13M:9F, black; sut/sut, *n* = 14M:10F, blue]. Spatial learning is measured as time to the escape chamber (i.e., primary latency) across days and graphed as mean ± SEM per each day. Data were analyzed via MMRM ANOVA followed by Dunnett’s multiple comparisons test. The percentage of direct [light gray or light blue], serial [gray or blue] and random [white with black or blue borders] strategies used per each training days are graphed. Differences in strategy used between groups or days were determined by Fisher’s exact test. No within genotype sex differences were found. **(A)** Young sut/sut male mice took longer to find the escape chamber as compared to +/+ , but both genotypes improved their performance over time: F_(1_, _24)_ = 8.628, *p* = 0.0072, genotype; F_(2_._206_, _50_._06)_ = 15.13, *p* < 0.0001, training days; F_(3_, _68)_ = 0.4644, *p* = 0.7081, interaction, performing significantly better on the last day (d1 vs d4: *p = 0.0406 for +/+ , ^#^*p* = 0.0031 for sut/sut). **(B)** Young female mice improved their performance on the maze over time: F_(2_._669_, _56_._06)_ = 15.58, *p* < 0.0001, training days; F_(1_, _21)_ = 0.51578, *p* = 0.4806, genotype; F_(3_, _63)_ = 1.690, *p* = 0.1782, interaction, and performed better on the last day (d1 vs d4: *p < 0.0001 for +/+ , ^#^*p* = 0.0090 for sut/sut). **(C)** Young male mice adopted more effective (direct + serial) search strategy with training regardless of genotype (*d1 vs d4: *p* < 0.0001 for both +/+ and sut/sut). However, while +/+ male mice fully abandoned the use of a random strategy by day 4, sut/sut mice did not (^#^*p* = 0.0177 for d4). **(D)** Young female mice adopted more effective search strategies (direct + serial) across training days regardless of genotype (*d1 vs d4: *p* < 0.0001 for +/+ ; *p* = 0.0204 sut/sut). No genotype differences in strategy utilization by day 4 was found in female mice (p = 0.5757). **(E)** A main effect of training was present in adult male mice: F_(1_._613_, _37_._64)_ = 5.313, *p* = 0.0137, training days; F_(1_, _25)_ = 3.452, *p* = 0.0750, genotype; F_(3_, _70)_ = 0.7608, *p* = 0.5198 interaction. *Post hoc* analyses: adult +/+ mice improved their performance (*d1 vs d4: *p* = 0.0270) whereas adult sut/sut mice did not (d1 vs d4: p = 0.6446). Escape latency improved with age regardless of genotype: +/+ : F_(1_, _12)_ = 9.204, *p* = 0.0104 for age; sut/sut: F_(1_,_14)_ = 19.23, *p* = 0.0006 for age. **(F)** A main effect of training was present in adult females: F_(2_._728_, _50_._01)_ = 4.806, *p* = 0.0064, training days; F_(1_, _21)_ = 0.1057, *p* = 0.7483, genotype; F_(3_, _55)_ = 1.548, *p* = 0.2124, interaction]. *Post hoc* analyses: adult +/+ mice improved their performance (*d1 vs d4: *p* = 0.0431) whereas adult sut/sut mice did not (d1 vs d4: *p* = 0.1818). Escape latency improved with age regardless of genotype: +/+ : F_(1_, _12)_ = 28.72, *p* = 0.0002 for age; sut/sut: F_(1_,_9)_ = 12.72, *p* = 0.0061 for age. **(G)** Male mice adopted more effective strategies (direct + serial) across training days regardless of genotype (*d1 vs d4: *p* = 0.0003 for +/+ ; *p* = 0.0008 sut/sut). **(H)** Female mice also adapted more effective strategies (direct + serial) across training days regardless of genotype (*d1 vs d4: *p* = 0.0030 for +/+ ; *p* = 0.0064 for sut/sut). Adult female +/+ mice persisted in their use of a random strategy as compared to young +/+ females (^#^d4: young vs adult: *p* = 0.0427) and as compared to adult +/+ male mice (^$^d4: *p* = 0.0427). No differences between adult +/+ and sut/sut female mice in their use of strategies was uncovered (d4: *p* = 0.1211).

Escape performance improved in all male mice irrespective of genotype. However, young SLC7A11^sut/sut^ male took longer to escape the maze than their age- and sex-matched counterparts (Figure 8A). During our analysis of search strategies employed, we found that on day 1 young male mice of both genotypes utilized a combination of different strategies, with random searching predominating. By day 4, there was a notable shift toward the use of more efficient strategies, defined as direct plus serial searching. In the young SLC7A11*^+/+^*, the utilization of these efficient strategies increased to 100%. In contrast, SLC7A11^sut/sut^ male mice exhibited a lower adoption of efficient strategies reaching only 84% of the trials. This disparity in strategy usage between the two genotypes was statistically significant (Figure 8C). As with males, young females of both genotypes resolved the maze on day one with significantly shorter latencies by the end of the training period (Figure 8B). Like males, young females initially utilized a mix of search strategies to find the escape chamber, but by day 4 more efficient strategies were employed, in this case, to a similar extent by both genotypes — 97 vs 93% of trials for SLC7A11*^+/+^* and SLC7A11^sut/sut^ mice, respectively (Figure 8D). We did not find any genotype differences in overall speed or distance traveled in either male or female mice (data not shown).

Interestingly, adult mice of both sexes outperformed their younger selves by resolving the maze faster. While MMRM ANOVA revealed only a main effect of training, *post hoc* analyses revealed that adult SLC7A11*^+/+^* mice improved their performance whereas adult SLC7A11^sut/sut^ mice did not (Figures 8E, F). No genotype differences in overall speed and distance traveled by either male or female adult mice was found (data not shown). With respect to search strategy, adult mice initially used both random and non-random search strategies at similar percentages to when they were 2 months of age. By day 4, a more efficient serial strategy was employed by adult males (97 and 90% of trials for SLC7A11*^+/+^* and SLC7A11^sut/sut^ mice, respectively) and adult females (82 and 96% of trails for SLC7A11*^+/+^* and SLC7A11^sut/sut^ mice, respectively) (Figures 8G, H), indicating the mice were all able to learn to adopt a more efficient strategy. Interestingly, a non-random strategy was used less frequently when female SLC7A11*^+/+^* aged, an effect not seen with mice in any other group. Additionally, a sex difference was revealed in the strategies used to escape the maze between male and female SLC7A11*^+/+^* adult mice with males employing the serial strategy in 97% of trials vs 82% of trials for females (Figures 8G, H).

### 3.8. Acoustic startle response (ASR), prepulse inhibition (PPI) and habituation

**FIGURE 9:**
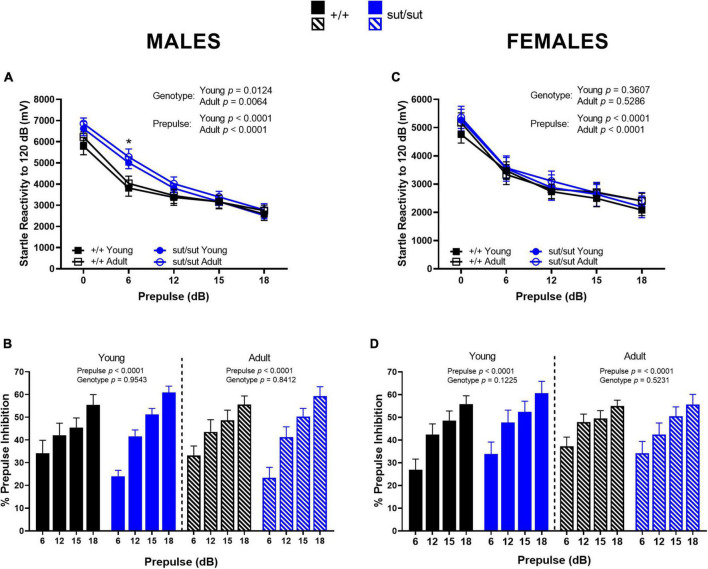
Acoustic startle reactivity and percent prepulse inhibition. Male **(A,B)** or Female **(C,D)** SLC7A11^+/+^ (+ / + , n = 13M:14F, black) and SLC7A11^sut/sut^ (sut/sut, n = 15M:10F, blue) littermates were tested at 2 (young) and at 6 months (adult). ASR and PPI are graphed as mean ± SEM. Data were analyzed within each age group by two-way ANOVA followed by Bonferroni’s multiple comparisons test. Sex Differences: Female mice have an overall lower startle reactivity when compared to age- and genotype-matched male mice [young: F_(1_,_125)_ = 10.16, p = 0.0018 for sex for +/+ ; F_(1_,_115)_ = 20.86, *p* < 0.0001 for sex for sut/sut; adult: F_(1_,_130)_ = 9.820, p = 0.0021 for sex for +/+ ; F_(1_,_115)_ = 24.44, *p* < 0.0001 for sex for sut/sut]. No within genotype sex differences were found in PPI. **(A)** Mean startle reactivity is higher in sut/sut male mice, regardless of age, as compared to +/+ male mice when the prepulse delivered is below 15dB. *Young:* F_(4_, _130)_ = 6.424, *p* = 0.0124, genotype; F_(4_, _130)_ = 44.94, *p* < 0.0001, prepulse; F_(4_, _130)_ = 1.633, *p* = 0.1698, interaction. *Adult:* F_(4_, _135)_ = 7.680, *p* = 0.0064, genotype; F_(4_, _135)_ = 45.86, *p* < 0.0001, prepulse; F_(4_, _135)_ = 1.118, *p* = 0.3506, interaction. This difference is significant at the 6 dB prepulse *(*p* = 0.0267 for young; *p* = 0. 0253 for adult). **(B)** PPI significantly increased with prepulse intensity regardless of genotype in male mice: *Young:* F_(3_,_108)_ = 19.33, *p* < 0.0001, prepulse; F_(3_,_108)_ = 0.0033, *p* = 0.9543, genotype; F_(1_, _108)_ = 1.672 *p* = 0.1772, interaction. *Adult:* F_(3_,_108)_ = 20.86, *p* < 0.0001, prepulse; F_(1_,_108)_ = 0.0404, *p* = 0.8412, genotype; F_(3_, _108)_ = 1.685 *p* = 0.1745, interaction]. Young vs adult: +/+ : F_(1_, _52)_ = 0.3196, *p* = 0.5743 for age; sut/sut: F_(1_, _56)_ = 3.583, *p* = 0.0636 for age. **(C)** Mean startle reactivity did not differ between genotype at either age tested in female mice: *Young:* F_(4_, _110)_ = 0.8425, *p* = 0.3607, genotype; F_(4_, _110)_ = 21.91, *p* < 0.0001, prepulse; F_(4_, _110)_ = 0.1343, *p* = 0.9694, interaction. *Adult:* F_(1_,_110)_ = 0.3995, *p* = 0.5286, genotype; F_(4_, _110)_ = 22.05, *p* < 0.0001, prepulse; F_(4_, _110)_ = 0.0856, p = 0.9867, interaction. **(D)** PPI significantly increased with prepulse intensity regardless of genotype in female mice: *Young:* F_(3_,_88)_ = 12.46, *p* < 0.0001, prepulse; F_(1_,_88_) = 2.432, *p* = 0.1225, genotype; F_(3_, _88)_ = 0.0568, *p* = 0.9821, interaction. *Adult:* F_(3_,_88)_ = 8.638, *p* < 0.0001, prepulse; F_(1_,_88)_ = 0.4111, *p* = 0.5231, genotype; F_(3_, _88)_ = 0.3264, *p* = 0.8063, interaction. Young vs adult: +/+ : F_(1_, _52)_ = 3.705, *p* = 0.0597 for age; sut/sut: F_(1_, _36)_ = 1.268, *p* = 0.2676 for age.

*ASR and PPI.* In male mice, we found a significant main effect of genotype and prepulse in the ASR at both ages tested. *Post hoc* analyses revealed no genotype difference in ASR at 120 dB at either 2 or 6 months of age (Figure 9A). However, SLC7A11^sut/sut^ male mice at both ages demonstrated a significantly higher startle response to the lowest prepulse administered (6dB) when compared to their age-matched SLC7A11*^+/+^* male littermates, indicating that male mice lacking Sx_c_^–^ do not filter small amplitude stimuli as efficiently as do wildtype mice (Figure 9A). Consistent with this, PPI was reduced to a lesser extent at the 6db prepulse sound in SLC7A11^sut/sut^ male mice: 24.0 vs 34.2% in young SLC7A11^sut/sut^ and SLC7A11*^+/+^* respectively (*p* = 0.0778; Mann Whitney U), and 23.3 vs 33.2% in adult SLC7A11^sut/sut^ and SLC7A11*^+/+^* respectively (*p* = 0.0923; Mann Whitney U) (Figure 9B). Despite this, the overall level of PPI of the ASR analyzed across all four stimuli was not significantly disrupted by loss of Sx_c_^–^ at either age or by age within genotype (Figure 9B).

In female mice, a main effect of prepulse, but not genotype, was found in the ASR at both ages tested (Figure 9C). Likewise, loss of Sx_c_^–^did not disrupt PPI of the ASR at either age. Interestingly, the percent PPI increased with age in female SLC7A11*^+/+^* mice, an effect primarily driven by a change in response to the 6 dB prepulse (*p* = 0.0469). This enhancement was not evident in female SLC7A11^sut/sut^ mice (Figure 9D).

**FIGURE 10:**
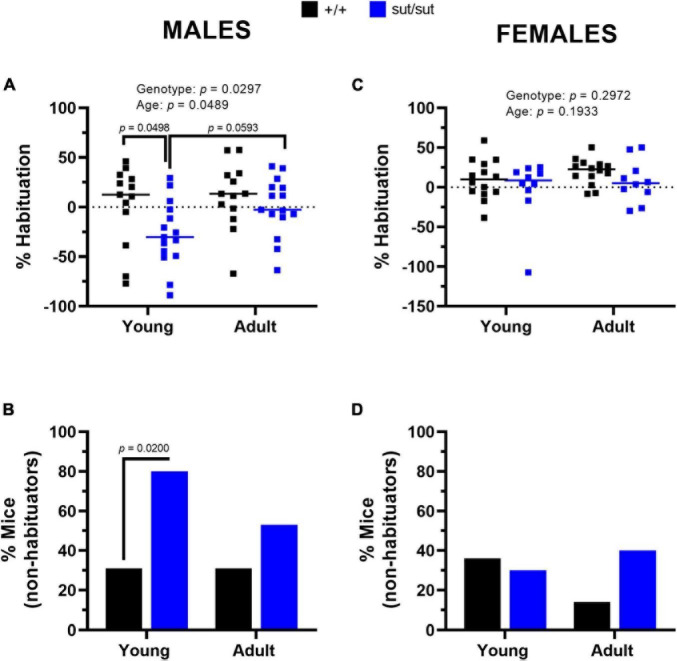
Percent habituation. Male **(A,B)** or Female **(C,D)** SLC7A11^+/+^ (+ / + , n = 13M:14F, black) and SLC7A11^sut/sut^ (sut/sut, n = 15M:10F, blue) littermates were tested at 2 (young) and at 6 months (adult). Each data point representing an individual animal is graphed with the median percent habituation (100 x [(mean startle amplitude on the first 6 trials - mean startle amplitude on the last 6 trials)/mean startle amplitude on the first 6 trials]) reflected by the horizontal line. Statistical analyses were performed on the habituation score [(mean startle amplitude on the last 6 trials)/(mean startle amplitude on the first 6 trials)] using MMRM ANOVA followed by Bonferroni’s multiple comparisons test. The number of non-habituating mice graphed as a percentage was analyzed using the Fisher’s exact test. *Sex Differences:* Percent habituation is disrupted in sut/sut male mice as compared to sut/sut female mice [F _(1_,_48)_ = 4.301, *p* = 0.0435 for sex]. No sex differences were found in +/+ mice [F_(1_,_48)_ = 0.7447, *p* = 0.3925 for sex]. **(A)** *Habituation in males*: F_(1_, _52)_ = 4.998, *p* = 0.0297, genotype; F_(1_, _52)_ = 4.066, *p* = 0.0489, age; F_(1_, _52)_ = 1.064, *p* = 0.3071, interaction. (+ / + vs sut/sut: *p* = 0.0498 for young, *p* = 0.7967 for adult) (young vs adult: *p* > 0.9999 for +/+ , *p* = 0.0593 for sut/sut). **(B)** Male *non-habituator mice*. Genotype: +/+ vs sut/sut (*p* = 0.0200 for young, *p* = 0.2761 for adult); Age: young vs adult (*p* > 0.9999 for +/+ , *p* = 0.2451 for sut/sut). **(C)** *Habituation in females*: F_(1_, _22)_ = 1.140, *p* = 0.2972, genotype; F_(1_, _22)_ = 1.801 *p* = 0.1933, age; F_(1_, _22)_ = 0.0386, *p* = 0.8461, interaction. (+ / + vs sut/sut: *p* > 0.9999 for young, *p* = 0.7085 for adult) (young vs adult: *p* = 0.5362 for +/+ , *p* = 0.8894 for sut/sut). **(D)** Female *non-habituator mice*. Genotype: +/+ vs sut/sut (*p* > 0.9999 for young, *p* = 0.1924 for adult); Age: young vs adult (*p* = 0.3845 for +/+ , *p* > 0.9999 for sut/sut).

*Startle Habituation.* Inter-individual differences in habituation were much more pronounced in male mice of either genotype than female mice of either genotype (Figures 10A, C). Strikingly, in the young males, 80% of the SLC7A11^sut/sut^ mice did not habituate as compared to just 30% of SLC7A11*^+/+^* mice, a difference that was significant (Figure 10B). A lack of habituation reflects an enhanced startle response at the end of the paradigm as compared to the beginning (i.e., sensitization), resulting in an overall negative% habituation in the SLC7A11^sut/sut^ male mice (*Young*: −30.3% median; *Adult*: −2.8% median). The negative% habituation in the SLC7A11^sut/sut^ male mice was significantly different from the SLC7A11*^+/+^* male mice when tested at 2 months, but not later at 6 months (Figure 10A). These results indicate that young SLC7A11^sut/sut^ male mice sensitized to the startle stimulus, but with age their response normalized to their age matched wildtype littermates. In the female mice, there were no age- or genotype-dependent differences in the percent of mice that habituated. Furthermore, the fraction of mice that did not habituate to the startle stimulus did not differ at either age (Figures 10C, D). Altogether, these results indicate that Sx_c_^–^ signaling is necessary for sensorimotor gating to adequately filter information and to regulate habituation to the acoustic startle stimulus in male mice only.

## 4. Discussion

Sx_c_^–^ — widely distributed in the CNS and primarily expressed on astrocytes under normal conditions (Pow, 2001; Ottestad-Hansen et al., 2018) — contributes to the function of numerous neurotransmitter systems by releasing glutamate into the extracellular space (Cartmell and Schoepp, 2000; Baker et al., 2002b; Kullmann and Semyanov, 2002; Zhang and Sulzer, 2003). Perturbations in the interactions between different neurotransmitter systems can result in alterations in behavioral responses (Sarter et al., 2007; Teleanu et al., 2022). Indeed, previous behavioral characterization of xCT ^–/–^ male mice maintained in a C57Bl/6 background, using some, but not all, of the same test utilized herein, provide evidence for the involvement of Sx_c_^–^ in regulating anxiety and depression (Bentea et al., 2015) and spatial working memory (De Bundel et al., 2011) but not motor coordination (Bentea et al., 2021). To further investigate the impact of Sx_c_^–^ activity, we conducted a comprehensive set of motor, sensory, and social behavioral tests (Figure 1) on male and female SLC7A11^+/+^ and SLC7A11^sut/sut^ littermate mice at 2 and 6 months of age to determine whether loss of Sx_c_^–^ activity arising from a spontaneous mutation in *SLC7A11* in the C3H/HeSnJ strain of mice similarly altered these behaviors and others, and if so, whether this occurred in a sex- and/or age-dependent manner. Considering that genetic background can influence the expression of other genes and modifiers, potentially modifying the observed phenotype, convergence of results from different strains of Sx_c_^–^ mutants would provide compelling evidence for the critical role of Sx_c_^–^ dysfunction in shaping the observed behavioral phenotypes.

To ensure that gross motor behavior did not confound subsequent assessments, we first evaluated motor performance using the rotor rod (Figure 2). Notably, our results demonstrate that Sx_c_^–^ had no discernable effect on motor performance, in agreement with previous studies in SLC7A11^sut/sut^ and xCT ^–/–^ male and female mice (McCullagh and Featherstone, 2014; Bentea et al., 2021). The consistency of these outcomes across different mouse models reinforces the conclusion that Sx_c_^–^ plays no role in motor performance. Our study did, however, reveal an age-dependent decline in motor performance, which has been extensively documented previously, although the age in which this declination occurs can vary across different mouse strains (Barreto et al., 2010; Graber et al., 2013; Tung et al., 2014; Bentea et al., 2015). Our results show that C3H/HeSnJ mice exhibit a decline in motor performance by 6 months of age, regardless of sex.

Despite the findings on performance, our study is the first to reveal that Sx_c_^–^ signaling is essential for optimal motor learning and, intriguingly, its impact varies by sex. While no genotype difference in motor learning was observed in male mice, age was found to have a negative effect (Figure 2B). In contrast, SLC7A11^sut/sut^ females exhibited a significant reduction in motor learning compared to SLC7A11*^+/+^* females, regardless of age (Figure 2D). Interestingly, previous research has identified sex differences in cerebellar synaptic physiology, where female mice exhibit increased rates of spontaneous firing and enhanced excitation of metabotropic glutamate receptors (mGluRs) compared to males (Mercer et al., 2016). Notably, activation of mGluRs — important for regulating excitatory neurotransmission — relies in part on glutamate release by Sx_c_^–^ (Baker et al., 2002a; Bridges et al., 2012). Sx_c_^–^-released glutamate also directly activates non-NMDA glutamate receptors in the Purkinje cells of the cerebellum (Warr et al., 1999). Thus, it is intriguing to speculate that loss of Sx_c_^–^ may disproportionally impair cerebellar glutamatergic neurotransmission in females compared to males, potentially leading to their observed deficits in motor learning. Additionally, it would be interesting to investigate whether there is sex- and/or age-dependent decline in cerebellar plasticity in C3H mice as previous research has demonstrated reductions in cerebellar long term depression (LTD) in CBA mice between the ages of 4 and 8 months, an effect accompanied by behavioral impairment (Woodruff-Pak et al., 2010).

Reduced mGluR signaling, possibly through dysregulation of Sx_c_^–^, has also been linked to neuropsychiatric disorders characterized by anxiety and social deficits (e.g., autism spectrum disorder (ASD) and schizophrenia) (Chen et al., 2014; Huang et al., 2018; Crupi et al., 2019). For instance, the increased anxiety and deficits in social interaction found in a rat model of ASD, could be ameliorated by treatment with N-acetlycysteine (NAC), a cystine prodrug that increases extracellular glutamate levels by stimulating Sx_c_^–^, occurring in an mGluR-dependent manner (Chen et al., 2014). Additionally, in organotypic brain slice cultures, genetic or pharmacological inhibition of RGS4 (Regulator of G-protein Signaling 4), a putative susceptibility gene for schizophrenia, resulted in a decrease in xCT expression and Sx_c_^–^ activity, which was accompanied by reduced glutamate levels, the latter of which was ameliorated by NAC (Huang et al., 2018). NAC also ameliorated the schizophreniform-like symptoms observed when RGS4 was knocked down in the prefrontal cortex of mice (Huang et al., 2018).

To assess anxiety-like behavior and sociability in our mice, we used ethologically relevant exploration-based tests [open field, EPM and social interaction]. In the open field test, we found that male SLC7A11^sut/sut^ mice spent greater time in the center of the open space at 6 months of age than their age- and sex-matched littermate SLC7A11*^+/+^* mice (Figure 3D). This aligns with findings in xCT ^–/–^ mice tested at a similar age ranges (Bentea et al., 2015) but differs from McCullagh and Featherstone, who reported no change in center time in SLC7A11^sut/sut^ male mice examined once when 3–4 months old (McCullagh and Featherstone, 2014). However, these latter results were compared to non-littermate wildtype mice, which are not considered an appropriate control. While an increase in center time is often interpreted as a reduction in open-space anxiety, we propose that adult, but not young, male SLC7A11^sut/sut^ mice are instead hyperactive. This idea is supported by our findings that adult male SLC7A11^sut/sut^ travel increased distances in the open field (Figure 3), have increased rearing behavior (Figure 4), and show no evidence of open-space anxiety in the EPM, although this was observed when they were younger (Figure 5A). Finally, social interaction (preference for mouse over object) was normal in male SLC7A11^sut/sut^ mice regardless of age (Figure 6). The increased locomotion, normal sociability and anxiety traits found in SLC7A11^sut/sut^ male mice in our study contrasts somewhat with previous findings in xCT ^–/–^ mice (2–3.5 months) (Bentea et al., 2015; Bentea et al., 2021). Specifically, Bentea et al. (2015) report that loss of Sx_c_^–^ has no effect on locomotion, but creates an anxiolytic effect, with young male xCT ^–/–^ mice also exhibiting reduced sociability (Bentea et al., 2021). Further investigation is needed to determine if these differences can be attributed to the type of mutation (transgenic vs. spontaneous) and/or the influence of genetic background — C56BL/6 vs. C3H/HeSnJ (Coleman and Hummel, 1973; Spencer et al., 2011). Additionally, differences in age and study design could be important. Specifically, hyperactivity did not emerge in male Sx_c_^–^ nulls in our study until 6 months of age. Additionally, retrospective design, in which different cohorts of mice were tested in different paradigms, was utilized to test for anxiety in the xCT ^–/–^ male mice (Bentea et al., 2015). As test history affects behavioral outcomes (Voikar et al., 2004), this could lead to the inconsistency between studies. Moreover, while there was a significant reduction in the time that xCT ^–/–^ male mice spent with a novel mouse, they still showed a preference for the mouse over the object (Bentea et al., 2021). Finally, Bentea et al. (2021) used a light/dark paradigm to assess for anxiety-like behavior whereas we utilized the EPM. The light/dark paradigm could not be replicated in C3H/HeSnJ mice due to their visual impairment.

In contrast to adult males, no hyperactivity was detected in adult female SLC7A11^sut/sut^ mice, who spent similar amounts of time in the center, rearing and locomoting in the open field as did their age- and sex-matched SLC7A11^+/+^ littermate mice (Figures 3, 4). However, female SLC7A11^sut/sut^ mice at both ages displayed open-space anxiety as demonstrated by a reduction in both the number of entries and time spent in the open arms of an EPM when compared to their age- and sex-matched SLC7A11*^+/+^* littermate controls (Figure 5). Furthermore, at both ages, female SLC7A11^sut/sut^ mice exhibited social deficits showing no preference for the novel mouse as compared to the novel object (Figure 6). Although vision of our mice is impaired, intact olfaction (Supplementary Figure 1) suggests that loss of chemosensory cues were not responsible for this atypical social behavior (Ryan et al., 2008). Increased anxiety of female SLC7A11^sut/sut^ is also unlikely to account for deficits in sociability, as exploratory behavior in test chambers did not differ between groups (Supplementary Figure 2). It is worth mentioning that a recent study reported that social preference in C57BL/6 female mice may be influenced by their estrous cycle (Chari et al., 2020). However, it is important to highlight that Chari et al. (2020) used a paradigm where the experimental mouse chose between a novel mouse and a familiar object, making it challenging to fully differentiate sociability versus novel exploration. In contrast, our 3-chamber test involves the choice between a novel stranger and a novel object, allowing us to control for novelty driven exploration (Yang et al., 2011). Furthermore, estrus cycle can be affected, either prolonged or suppressed, in grouped housed mice (4–6 per cage) as well as in blind mice whether housed in groups or individually (Whitten, 1959; Champlin, 1971). Given that our mice are blind and housed 3–5 per cage, it is plausible that they may show similar irregularities perhaps making estrous in our mouse model irrelevant.

The behavioral phenotype of female SLC7A11^sut/sut^ mice is especially intriguing as it closely resembles that of mouse models of ASD that show a reduction in sociability, increased anxiety-like behavior (Peça et al., 2011; Yang et al., 2021; Singla et al., 2022) and deficits in motor learning (Giza et al., 2010; Chévere-Torres et al., 2012; Tsai et al., 2018). These behavioral manifestations are thought to arise from an imbalance in excitation/inhibition (E/I) within the neural circuits involving the medial prefrontal cortex (mPFC), the amygdala and ventral hippocampus (Bateup et al., 2013; Allsop et al., 2014; Felix-Ortiz et al., 2016; Yang et al., 2021), as well as deficits in cerebellar transmission and function (Giza et al., 2010; Chévere-Torres et al., 2012; Tsai et al., 2018). Notably, our lab has previously demonstrated that E/I balance is impaired in SLC7A11^sut/sut^ mice of both sexes (Sears et al., 2021). We also reported that specific morphological abnormalities are present in female SLC7A11^sut/sut^ mice as compared to SLC7A11*^+/+^* female littermates, including a reduction in the size of the corpus callosum and a reduced soma size of Layer 5 pyramidal neurons of the primary motor cortex (Sears et al., 2021). These morphological abnormalities have been reported in ASD mouse models — including C58/J (idiopathic autism), SHANK3 mutants (genetic factors) and Mecp2-null (Rett Syndrome) — as well as in humans with ASD (children and adults) (Chen et al., 2001; Frazier and Hardan, 2009; Wang et al., 2013; Ellegood and Crawley, 2015; Wegiel et al., 2015; Vitrac et al., 2020; Baron-Mendoza et al., 2021). Importantly, whole exome sequencing identified a missense variation in *SLC7A11* in a family with ASD siblings, although the specific impact of this variation is unclear (Egawa et al., 2015). Additionally, there was a strong linkage signal on chromosome 4 in a region containing *SLC7A11* in families with at least one female affected (Schellenberg et al., 2006). This linkage finding is further supported by additional analyses conducted using the UCSC genome browser (Autism Genome Project Consortium et al., 2007). Finally, overexpression of the long coding RNA, RPS10P2-AS1, the functional element of rs4141463, a polymorphism associated with ASD, resulted in a decrease in *SLC7A11* expression in human progenitor cells (Bilinovich et al., 2019). The convergence of these behavioral, morphological, and genetic abnormalities suggests a potential association between impaired Sx_c_^–^ function and ASD-related pathophysiology in females that requires further investigation.

Interestingly, open-space anxiety and deficits in sociability emerge with age in SLC7A11*^+/+^* female mice (Figure 5, 6). The presence of age-dependent increases in anxiety-like behaviors appears to be strain-dependent as studies by Zambrana et al. (2007) and Joshi and Pratico (2011) did not observe such changes in C57BL/6 or CD-1 female mice using the EPM (Zambrana et al., 2007; Joshi and Pratico, 2011). In contrast, Frick et al. (2000) reported enhanced anxiety-like behavior in older C57BL/6NIA female, but not male, mice (Frick et al., 2000). Our findings in C3H/HeSnJ mice are consistent with this pattern, suggesting that both strain- and sex-specific factors may contribute to age-related anxiety and social behavior.

In addition to anxiety-like behavior and sociability impairments alterations in other cognitive domains, including memory (Stearns et al., 2007; Brielmaier et al., 2012; Saurabh et al., 2013; Hara et al., 2016) and sensorimotor gating (Brielmaier et al., 2012; Samaco et al., 2013; Moy et al., 2014), have also been documented in mouse models of ASD. Herein, we investigated the role of Sx_c_^–^ in recognition and spatial memory and sensorimotor gating by comparing the performance of age- and sex-matched SLC7A11^sut/sut^ and SLC7A11*^+/+^* mice using the NOR to assess the differentiation of novel vs familiar objects, the Barnes maze to measure escape latency and search strategies, and the ASR and PPI of the ASR to investigate sensorimotor gating.

Notably, all mice, irrespective of their genotype, sex, or age, exhibited a positive NOR discrimination index (Figures 7B, D), indicating that recognition memory of Sx_c_^–^ null mice remained intact. These results are consistent with a previous study conducted in rats with a genetic disruption of Sx_c_^–^ (Hess et al., 2023). However, our investigation using the Barnes maze revealed disruptions in certain aspects of spatial memory (Figure 8). Specifically, while young SLC7A11^sut/sut^ male mice were able to solve the maze, they took longer to find the escape chamber than SLC7A11^+/+^ male mice, which is likely, in part, due to their failure to abandon the use of a random strategy for a more efficient one as compared to age-matched SLC7A11*^+/+^* littermates. Similarly, spatial reference memory was largely intact in young male xCT ^–/–^ mice, but they too were less efficient at learning, in this case, the hidden platform water maze task (De Bundel et al., 2011). In contrast, male adult SLC7A11^sut/sut^ mice were able to navigate the Barnes maze effectively, similar to the adult SLC7A11*^+/+^* mice, which agrees with a previous finding in xCT *^–/–^* mice (3–4 months old males) (Verbruggen et al., 2022). Notably, xCT *^–/–^* mice switched to a predominant direct strategy likely due to the fact that their visual acuity is intact (Verbruggen et al., 2022), while C3H/HeSnJ mice adopted a serial strategy thus effectively compensating for their visual impairment allowing for efficient location of the escape hole. The consistency of these outcomes across two Sx_c_^–^ null mouse models reinforces the conclusion that Sx_c_^–^ affects spatial reference memory task performance in young but not adult male mice. Female SLC7A11^sut/sut^ mice did not display any memory deficits at either age. Thus, there is an interesting interplay of factors — Sx_c_^–^, age and sex — that influence spatial memory and strategy selection.

Synaptic plasticity in the hippocampus, in the form of LTP and LTD, plays a pivotal role in memory processing and consolidation (Neves et al., 2008; Zemla and Basu, 2017; Abraham et al., 2019). The recognition memory circuit is complex, comprising several connections between the hippocampus and the PFC and the perirhinal cortex (PRC) (Chao et al., 2022). Recent research has revealed that LTP disruption within the PRC, rather than the hippocampus, was associated with impaired recognition memory (Outram et al., 2022), whereas hippocampal LTP disruption was linked to diminished spatial memory (Li et al., 2002; Yujiao et al., 2019). Intriguingly, hippocampal LTP disruption has been reported in 3 months old male SLC7A11^sut/sut^ mice (females not studied), which was accompanied by impairment in fear and passive avoidance memories (Li et al., 2012). In addition, SLC7A11^sut/sut^ mice show impairment in working memory, which is also hippocampus-dependent (McCullagh and Featherstone, 2014). Collectively, these results suggest that Sx_c_^–^contributes to the formation of associative, working, and spatial memories in male mice.

Interestingly, age influenced both recognition and spatial memory formation occurring in a sex-dependent manner. In male mice, the discrimination index increased at 6 months of age, indicating improved recognition memory. Moreover, mice of both sexes at this age exhibited reduced latency to find the escape hole in the Barnes maze, indicative of improved spatial memory. These observations in males may be attributed to brain reorganization and changes in hippocampal connectivity that occur beyond sexual maturity (Hammelrath et al., 2016; Kerkenberg et al., 2021). On the other hand, the relatively steady discrimination index in females across ages may be linked to sex differences in timing of brain maturation, as seen in C57BL/6J mice (Qiu et al., 2018). Alternatively, the shorter navigation time observed in the Barnes maze at 6 months in both sexes, could be attributed to enhanced procedural memory as the mice previously experienced the maze at a younger age (Heimer-McGinn et al., 2020).

Sensorimotor gating is a critical process for preventing sensory overload to ensure normal information processing (Ioannidou et al., 2018; Gómez-Nieto et al., 2020). Since baseline startle response can be a confounding factor in interpreting PPI (Yee et al., 2005; Shoji and Miyakawa, 2018; Scarborough et al., 2019), we analyzed both PPI and the ASR individually. Interestingly, we observed intact PPI in all mice regardless of sex and genotype, indicating their ability to suppress a motor response after a prepulse preceded the startle stimulus (Figure 9). However, male SLC7A11^sut/sut^ exhibited a lower degree of the motor response suppression compared to male SLC7A11*^+/+^* mice, visible graphically by the decrease in steepness of the reactivity curve (Figure 9A). This suggests a decrease in filtering efficiency. A deficit in sensory filtering is also supported by our findings of negative habituation — that is sensitization — in SLC7A11^sut/sut^ male, occurring most prominently at 2 months of age (Figure 10). Sensitization reflects a state of heightened alertness (Groves and Thompson, 1970). In contrast, we did not find any genotype difference in habituation and ASR in female mice. However, ASR in female mice was significantly lower as compared to their genotype-matched male mice, consistent with previous results in both C57BL/6J and C3H strains (Plappert et al., 2005).

Given the presence of other phenotypes associated with ASD, we initially expected SLC7A11^sut/sut^ female mice to display deficits in PPI. However, our findings are consistent with other mouse models of ASD including SHANK3 mutants (associated with Phelan-McDermid syndrome) and Fmr1 mutants (associated with Fragile-X Syndrome), which also show no impairment in PPI (Jaramillo et al., 2016; Schaefer et al., 2017). Interestingly, similarly variability is observed in humans with ASD (Cheng et al., 2018). Therefore, impaired social behavior stands out as the primary hallmark shared among ASD mouse models, and it is often accompanied by associated symptoms such as anxiety (Kazdoba et al., 2016). It is noteworthy that impaired social behavior was not found in male SLC7A11^sut/sut^ mice. Instead, male SLC7A11^sut/sut^ mice displayed other behavioral phenotypes including a lower efficiency in sensory filtering, heightened anxiety, sensitization, and impaired spatial learning when young, along with emerging hyperactivity in adulthood. These behavioral traits are commonly associated with attention deficit hyperactivity disorder (ADHD) in ADHD mouse models (Jhang et al., 2017; Custodio et al., 2021) as well as in individuals with ADHD (Reimherr et al., 2017; Sommer et al., 2021). Interestingly, the observation of hyperactivity found exclusively in male SLC7A11^sut/sut^ mice aligns with the predominance of hyperactivity in male individuals diagnosed with ADHD (Williamson and Johnston, 2015). Finally, we note that genes associated with ADHD susceptibility, such as *DRD5* that encodes dopamine receptor 5 (Manor et al., 2004) and *SLC6A3* that encodes dopamine transporter 1 (Faraone et al., 2014), are both located in close proximity to the *SLC7A11* located on chromosome 4. This is particularly interesting given that basal levels of glutamate, which are maintained by Sx_c_^–^, are found to be altered by drugs that modify dopaminergic systems (Baker et al., 2003).

In summary, our findings suggest that Sx_c_^–^ signaling contributes to motor learning, typical social behavior, and regulation of anxiety levels in female mice, independent of age. Loss of Sx_c_^–^ function in female mice leads to behavioral phenotypes that resemble mouse models of ASD, as well as human ASD. Conversely, in male mice, Sx_c_^–^ contributes to normal locomotor activity, open-space anxiety, spatial learning, and sensorimotor gating in an age-dependent manner, with loss of function resulting in behavioral phenotypes that resemble mouse models of ADHD and human ADHD. These findings align with our previous observations of sexually dimorphic neuronal architecture and brain oxidation status in SLC7A11^sut/sut^ mice (Sosnoski et al., 2020; Sears et al., 2021). Taken together, these results highlight the potential importance of further investigating the role of *SLC7A11* in neurodevelopment.

## Data availability statement

The original contributions presented in this study are included in this article/Supplementary material, further inquiries can be directed to the corresponding author.

## Ethics statement

The animal study was approved by the Syracuse University Institutional Animal Care and Use Committee. The study was conducted in accordance with the local legislation and institutional requirements.

## Author contributions

CF and SH conceived and designed the experiments and wrote the manuscript. CF created the final figures with input from SH and performed the statistical analyses. SP conducted the experiments and performed the preliminary analysis of the data. All authors approved the submitted version.

## Supplementary material

Supplementary Figure 1 Odor-Induced c-fos mRNA Expression in the Olfactory Bulbs. Mice were exposed to a naïve cotton swab (grey bar, non-odor) or a swab containing peppermint (green bars, odor) for 30 min, after which they were sacrificed, and olfactory bulbs removed for analysis. Total RNA was isolated, reverse transcribed and c-fos and HPRT expression assessed by q-RT-PCR. Data are expressed as mean fold change in c-fos mRNA expression over non-odor of each respective genotype as represented by the horizontal line of each bar. Individual mice are represented by open (males) or closed (females) circles. An asterisk (*) denotes statistically significant differences between odor and non-odor exposed mice as determined by two-way ANOVA followed by Bonferroni’s multiple comparison test of the geometric means: F_(1,36)_ = 51.39, *p* < 0.0001, odor; F_(1,36)_ = 1.332, *p* = 0.2561, genotype; F_(1,36)_ = 1.495 *p* = 0.5287, interaction (*p <* 0.0001 for +/+, *n* = 10 each*; p* = 0.0003 for sut/sut, *n* = 10–11).

Supplementary Figure 2 Chamber Time in the 3-Chamber Social Test. Male **(A)** or Female **(B)** SLC7A11*^+/+^* and SLC7A11*^sut/sut^* littermates were tested at 2 (young) [+/+, *n* = 11M:10F, black; sut/sut, *n* = 12M:8F, black] and at 6 months (adult) [+/+, *n* = 13M:9F, black; sut/sut, *n* = 13M:10F, blue]. Each data point representing an individual animal is graphed with the mean time in each chamber reflected by horizontal line of the bar. Median number of entries is reflected by the horizontal line of the box extending from the 25th to the 75th percentiles with the whiskers reaching the smallest and largest values. Data were analyzed within each age group by two-way ANOVA followed by Bonferroni’s multiple comparisons test. **(A)** Time in chambers — males. Young: F_(1,42)_ **=** 9.496, *p* **=** 0.0036, chamber; F_(1,42)_ **=** 0.439, *p* **=** 0.5108, genotype; F_(1,42)_ **=** 1.306, *p* **=** 0.2595, interaction (mouse chamber vs object chamber: *p* **=** 0.0129 for **+**/**+**, *p* **=** 0.1053 for sut/sut). Adult: F_(1,46)_ **=** 21.55, *p*** <** 0.0001, chamber; F_(1,46)_ **=** 0.0422, *p* **=** 0.8382, genotype; F_(1,46)_ **=** 2.388, *p* **=** 0.1292, interaction (mouse chamber vs object chamber: *p* **=** 0.002 for **+**/**+**, *p* **=** 0.0606 for sut/sut). **(B)** Time in chambers — females. Young: F_(1,32)_ **=** 5.753, *p* **=** 0.0225, chamber; F_(1,32)_ **=** 1.855e-007, *p* **=** 0.9997, genotype; F_(1,32)_ **=** 6.954, *p* **=** 0.0128, interaction (mouse chamber vs object chamber: *p* **=** 0.0013 for **+**/**+**, *p* **>** 0.9999 for sut/sut). Adult: F_(1, 34)_ **=** 1.627, *p* **=** 0.2107, chamber; F_(1, 34)_ **=** 0.0260, *p* **=** 0.8728, genotype; F_(1, 34)_ **=** 0.5464, *p* **=** 0.5464, interaction (mouse chamber vs object chamber: *p* **>** 0.9999 for **+**/**+**, *p* **=** 0.3597 for sut/sut). **(C)** *Number of entries — males*. *Young*: F_(1,42)_ = 1.881, *p* = 0.1775, chamber; F_(1,42)_ = 0.1859, *p* = 0.6685, genotype; F_(1,42)_ = 2.398, *p* = 0.1290, interaction (mouse chamber vs object chamber: *p* = 0.0993 for +/+, *p* > 0.9999 for sut/sut). *Adult*: F_(1,46)_ = 7364, *p* = 0.3953, chamber; F_(1,46)_ = 2.392, *p* = 0.1288, genotype; F_(1,46)_ = 0.3934, *p* = 0.5336, interaction (mouse chamber vs object chamber: *p* = 0.6169 for +/+, *p* > 0.9999 for sut/sut). **(D)** *Number of entries — females*. *Young*: F_(1,32)_ = 0.7980, *p* = 0.3784, chamber; F_(1,32)_ = 0.6251, *p* = 0.4350, genotype; F_(1,32)_ = 0.5499, *p* = 0.4637, interaction (mouse chamber vs object chamber: *p* = 0.4582 for +/+, *p* > 0.9999 for sut/sut). *Adult*: F_(1,34)_ = 0.3201, *p* = 0.5753, chamber; F_(1,34)_ = 0.4813, *p* = 0.4925, genotype; F_(1,34)_ = 0.5730, *p* = 0.4543, interaction (mouse chamber vs object chamber: *p* = 0.7368 for +/+, *p* > 0.9999 for sut/sut).
