# Supplementary figures and images for: Sex- and age-dependent contribution of System xc– to cognitive, sensory, and social behaviors revealed by comprehensive behavioral analyses of System xc– null mice
Source: Front Behav Neurosci. 2023 Aug 15;17:1238349. doi: 10.3389/fnbeh.2023.1238349 (PMC10462982; doi:10.3389/fnbeh.2023.1238349)

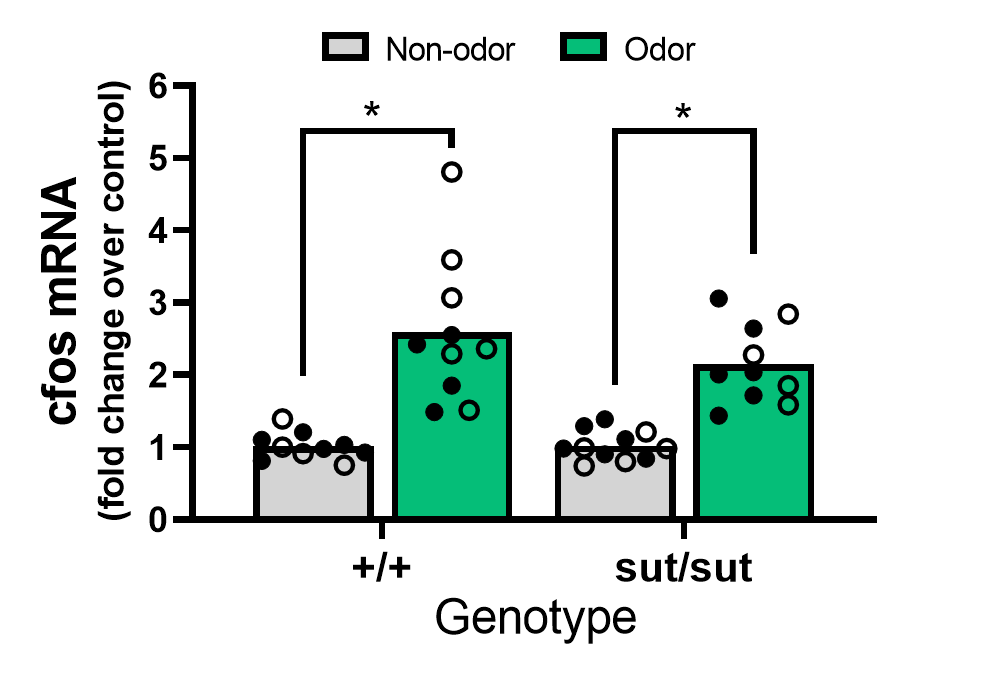

Supplement: Supplementary Figure 1 — Odor-Induced c-fos mRNA Expression in the Olfactory Bulbs. Mice were exposed to a naïve cotton swab (grey bar, non-odor) or a swab containing peppermint (green bars, odor) for 30 min, after which they were sacrificed, and olfactory bulbs removed for analysis. Total RNA was isolated, reverse transcribed and c-fos and HPRT expression assessed by q-RT-PCR. Data are expressed as mean fold change in c-fos mRNA expression over non-odor of each respective genotype as represented by the horizontal line of each bar. Individual mice are represented by open (males) or closed (females) circles. An asterisk (*) denotes statistically significant differences between odor and non-odor exposed mice as determined by two-way ANOVA followed by Bonferroni’s multiple comparison test of the geometric means: F(1,36) = 51.39, p < 0.0001, odor; F(1,36) = 1.332, p = 0.2561, genotype; F(1,36) = 1.495 p = 0.5287, interaction (p < 0.0001 for +/+, n = 10 each; p = 0.0003 for sut/sut, n = 10–11). [file Image_1.TIF]

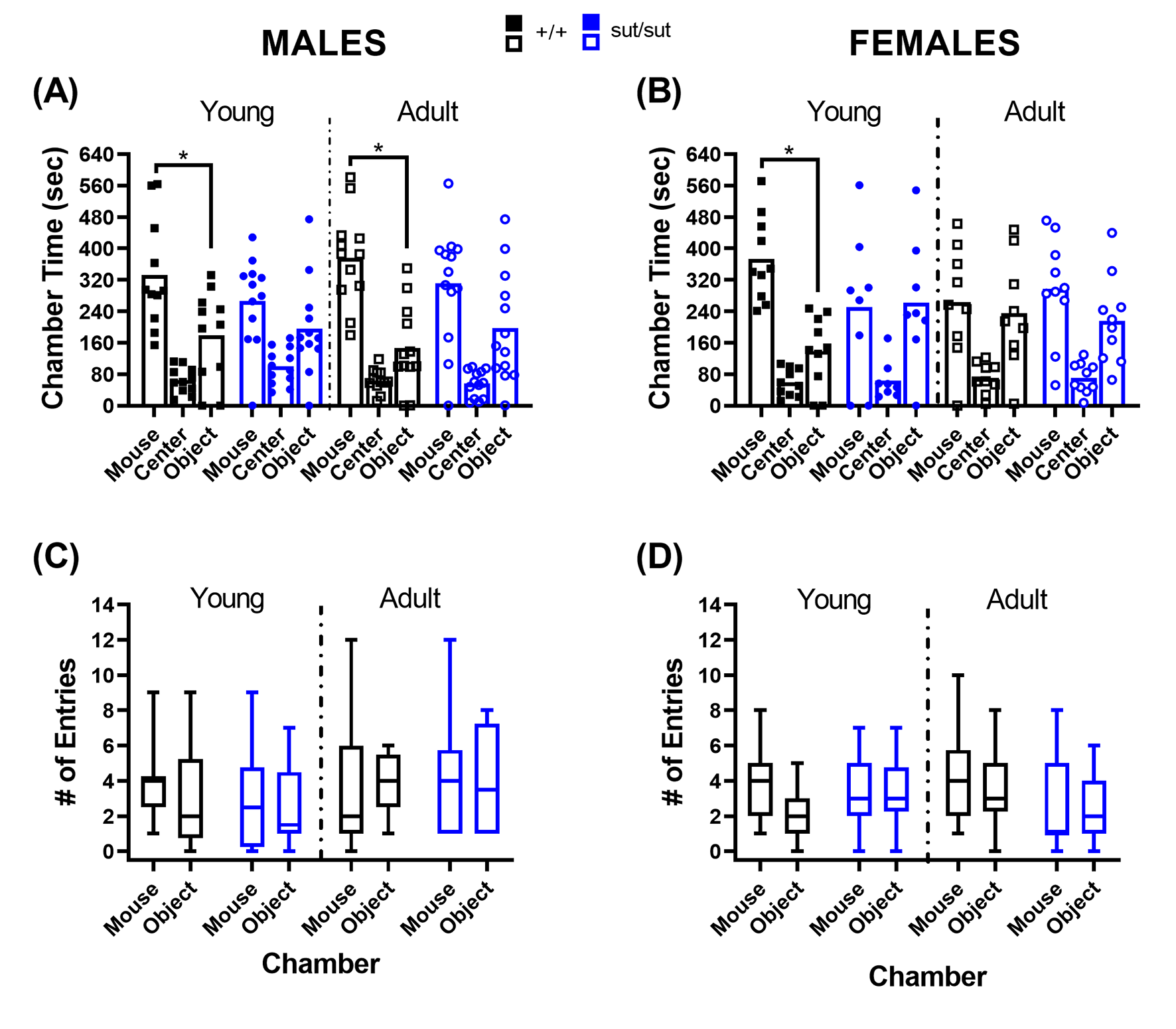

Supplement: Supplementary Figure 2 — Chamber Time in the 3-Chamber Social Test. Male (A) or Female (B) SLC7A11+/+ and SLC7A11sut/sut littermates were tested at 2 (young) [+/+, n = 11M:10F, black; sut/sut, n = 12M:8F, black] and at 6 months (adult) [+/+, n = 13M:9F, black; sut/sut, n = 13M:10F, blue]. Each data point representing an individual animal is graphed with the mean time in each chamber reflected by horizontal line of the bar. Median number of entries is reflected by the horizontal line of the box extending from the 25th to the 75th percentiles with the whiskers reaching the smallest and largest values. Data were analyzed within each age group by two-way ANOVA followed by Bonferroni’s multiple comparisons test. (A) Time in chambers — males. Young: F(1,42) = 9.496, p = 0.0036, chamber; F(1,42) = 0.439, p = 0.5108, genotype; F(1,42) = 1.306, p = 0.2595, interaction (mouse chamber vs object chamber: p = 0.0129 for +/+, p = 0.1053 for sut/sut). Adult: F(1,46) = 21.55, p < 0.0001, chamber; F(1,46) = 0.0422, p = 0.8382, genotype; F(1,46) = 2.388, p = 0.1292, interaction (mouse chamber vs object chamber: p = 0.002 for +/+, p = 0.0606 for sut/sut). (B) Time in chambers — females. Young: F(1,32) = 5.753, p = 0.0225, chamber; F(1,32) = 1.855e-007, p = 0.9997, genotype; F(1,32) = 6.954, p = 0.0128, interaction (mouse chamber vs object chamber: p = 0.0013 for +/+, p > 0.9999 for sut/sut). Adult: F(1, 34) = 1.627, p = 0.2107, chamber; F(1, 34) = 0.0260, p = 0.8728, genotype; F(1, 34) = 0.5464, p = 0.5464, interaction (mouse chamber vs object chamber: p > 0.9999 for +/+, p = 0.3597 for sut/sut). (C) Number of entries — males. Young: F(1,42) = 1.881, p = 0.1775, chamber; F(1,42) = 0.1859, p = 0.6685, genotype; F(1,42) = 2.398, p = 0.1290, interaction (mouse chamber vs object chamber: p = 0.0993 for +/+, p > 0.9999 for sut/sut). Adult: F(1,46) = 7364, p = 0.3953, chamber; F(1,46) = 2.392, p = 0.1288, genotype; F(1,46) = 0.3934, p = 0.5336, interaction (mouse chamber vs object chamber: p = 0.6169 for +/+, p > 0 [file Image_2.TIF]
